# A Promising Therapeutic Soy-Based Pickering Emulsion Gel Stabilized by a Multifunctional Microcrystalline Cellulose: Application in 3D Food Printing

**DOI:** 10.1021/acs.jafc.1c05644

**Published:** 2022-02-10

**Authors:** Mahdiyar Shahbazi, Henry Jäger, Rammile Ettelaie

**Affiliations:** † Institute of Food Technology, University of Natural Resources and Life Sciences (BOKU), Muthgasse 18, 1190 Vienna, Austria; ‡ Food Colloids and Bioprocessing Group, School of Food Science and Nutrition, University of Leeds, Leeds LS2 9JT, U.K.

**Keywords:** surface-active biopolymer, gallic acid, lauric arginate, antimicrobial properties, creep-recovery, frequency sweep, 3D printing performance, meat analogue, dynamic sensory evaluation

## Abstract

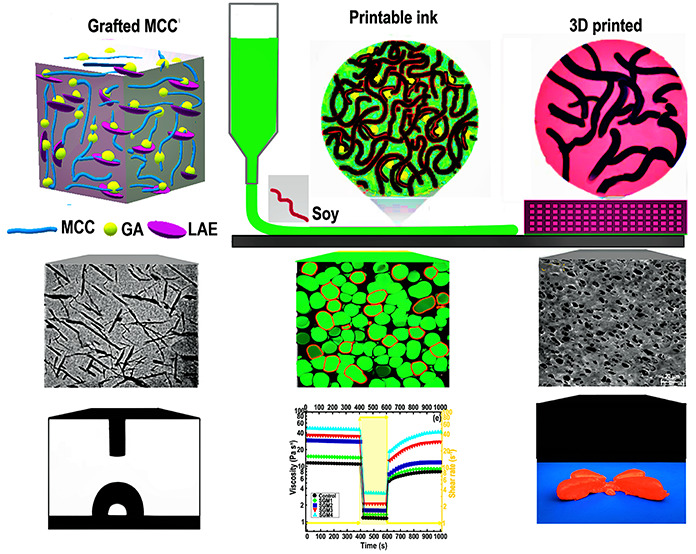

The feasible application of additive manufacturing in the food and pharmaceutical industries strongly depends on the development of highly stable inks with bioactive properties. Surface-modified microcrystalline cellulose (MCC) shows the potential of being a useful particulate (*i.e*., Pickering)-type emulsifier to stabilize emulsions. To attain desired therapeutic properties, MCC can also be tuned with cationic antimicrobial compounds to fabricate an antimicrobial printable ink. However, due to the formation of complex coacervates between the two, the Pickering emulsion is very susceptible to phase separation with an insufficient therapeutic effect. To address this drawback, we reported a green method to produce antioxidant and antimicrobial three-dimensional (3D)-printed objects, illustrated here using a printable ink based on a soy-based particulate-type emulsion gel stabilized by a surface-active MCC conjugate (micro-biosurfactant). A sustainable method for the modification of MCC is investigated by grafting gallic acid onto the MCC backbone, followed by *in situ* reacting *via* lauric arginate through Schiff-base formation and/or Michael-type addition. Our results show that the grafted micro-biosurfactant was more efficient in providing the necessary physical stability of soy-based emulsion gel. The grafted micro-biosurfactant produced a multifunctional ink with viscoelastic behavior, thixotropic property, and outstanding bioactivities. Following the 3D printing process, highly porous 3D structures with a more precise geometry were fabricated after addition of the micro-biosurfactant. Dynamic sensory evaluation showed that the micro-biosurfactant has a remarkable ability to improve the temporal perceptions of fibrousness and juiciness in printed meat analogue. The results of this study showed the possibility of the development of a therapeutic 3D-printed meat analogue with desired sensory properties, conceiving it as a promising meat analogue product.

## Introduction

Three-dimensional (3D) printing is considered to be the process of material assembly to construct 3D structures in a layer-by-layer manner.^1^ This technology can contribute to waste reduction and to the significant advancement of environmental sustainability on account of the decreased transportation, storage requirements, and the decentralization of material preparation.^2^ In this sense, the importance of selecting and characterizing the precise materials to obtain the desired structural and rheological properties, as a key requirement to attain an effective 3D printing process, can be clearly appreciated.^3^ Emulsion templating has been considered a common approach in additive manufacturing as it can offer proper pseudoplastic- and viscoelastic-type inks, with a reversible network that can lead to thixotropic behavior. Nevertheless, 3D printing using such emulsions is hampered by emulsion instability during application and its poor self-supporting feature. The latter presents numerous challenges in attaining an acceptable level of shape control. To overcome these obstacles, the application of particle-stabilized emulsions (Pickering emulsions) to the 3D printing process has attracted considerable attention in recent years.^4−6^ The main advantages of Pickering emulsions are their much-enhanced stability against droplet coalescence, arising from the almost irreversible absorption of particles at the interfaces, and their favorable flow characteristics.

Microcrystalline cellulose (MCC) is a highly crystalline material developed with the partial acid hydrolysis of α-cellulose, which has an extensive application in the pharmaceutical and food sectors. It shows a unique functional property as a thickening agent with rheology-modifying ability.^7^ However, unmodified MCC possesses a high surface charge density with a hydrophilic nature and hence offers a poor affinity for adsorption at oil–water interfaces. This makes MCC, in its native form, an unsuitable emulsifier for stabilizing O/W emulsions. Controlled physicochemical grafting modifications, through introducing hydrophobic groups, can remedy this shortcoming and confer innovative features to biopolymers without suppressing their desirable inherent properties.^8^ There exist several techniques for grafting onto the surface of a crystal. These include chemical grafting, high-energy radiation modification, plasma-induced technique, and grafting of polymeric chains.^9^ Despite numerous attempted techniques in hydrophobic modification, many remaining challenges still prevent the large-scale implementation of such MCC-based emulsifiers. The expensive modifier agents and environmental concerns they pose (regarding chemical process), steric hindrance (concerning polymer chains grafting), degradation of the polymeric backbone (*e.g*., in high-energy radiation-based techniques), and hydrophobic recovery (in the case of plasma treatment) are a few examples of such issues, limiting the wider commercial uses of hydrophobically modified MCC.^9,10^

In recent years, fabrication and application of antioxidant–biopolymer conjugates through the grafting of phenolic compounds onto the biomacromolecules backbone, have received significantly more interest.^11^ Reportedly, the grafting of the phenolic compounds offers biopolymers with desired flow properties and improved emulsifying features. Moreover, the grafted phenolic conjugates could also provide reinforced bioactivity *(i.e*., antioxidant activity) in comparison with their free-phenolic counterparts.^12−14^ Gallic acid (GA), as a secondary metabolite in plants, is recognized to provide therapeutic features such as antioxidant, anticancer, and anti-inflammatory properties.^12^ The GA grafted onto a biopolymer (for instance, MCC) can also be reacted with a biologically derived amphiphilic tag to enhance the emulsification stability. Particularly, the cationic surfactants were revealed to interact by a negatively charged biopolymer (*i.e*., MCC). Lauric arginate (LAE), as a biologically derived surface-active compound, is obtained from L-arginine and lauric acid, showing excellent therapeutic effects. There are several published works on the therapeutic effects of LAE against many bacteria in the microbiological growth medium^15^ and food matrices^16^ when used alone^17^ or in combination with other antimicrobials.^18^ As a valuable method, grafting LAE on the MCC can then decrease intra- or intermolecular hydrogen linkages, increasing the surface hydrophobicity of MCC and thus improving its emulsification property and biological activities of the pristine MCC.^11^

The accelerated growth of human population in the world and the subsequent impacts that this has on the consumption of natural resources are likely to lead to a lack of availability of proteins with a high biological significance. Recently, soy protein has received much attention as a promising resource in the development of meat alternatives due to its ability to provide suitable gel-like structures. However, soy protein alone is unable to offer a proper fibrous and layered matrix exhibited by common meat. In general, the soy-based emulsion is an unstable system, with soy protein having poor emulsifying capability compared to milk and other animal-derived proteins. When soy is employed to develop an O/W emulsion, only a small portion of this protein actually absorbs the droplets. This is due to its high molecular weight, compact globular structure, and low solubility. It was stated that the utilization of Pickering emulsions and emulsion gels in the formulation of meat analogues can improve its textural features.^19^ The gel-like structure produced with this multisystem can provide the essential consistency in the meat alternative products, ascribing the fibrous perception. On the other hand, the rising health awareness and increasing demand for reduced-fat meat alternatives drive researchers to focus on reducing the original fat in these products without the deterioration of the sensory quality. Once again, the application of Pickering emulsions and emulsion gels could offer a more effective technique for the replacement of the oil phase in the fat-substitute meat alternatives, while also preserving the textural and sensory features of the products.^19^

The main objective of the current work was to fabricate a reduced-fat 3D-printed meat analogue having a therapeutic property using the functionality of a modified bioactive MCC. To this end, a novel sustainable process for the modification of MCC was introduced. By grating GA onto the MCC backbone, an antioxidative MCC-*g*-GA was synthesized. This compound was *in situ* reacted with LAE through Schiff-base formation and/or Michael-type addition to offer therapeutic behavior to MCC-*g*-GA. Next, different levels of the grafted MCC conjugate were introduced to the soy-based emulsion to partially replace its oil phase. Finally, the prepared emulsion was processed with an extrusion-type printing system, and changes in the printing performance, morphology, and dynamic sensory features of 3D-printed objects were evaluated.

## Methods and Materials

### Material

The soy protein isolate (SPI) (moisture: 4.83%, fat: 0.32%, protein: 92.88%, ash: 3.40%, pH: 7.09, and viscosity of 1 wt % solution: 10.0 cP) was obtained from Archer Daniels Midland Company (ADM, Decatur, IL). Microcrystalline cellulose (MCC) Avicel PH-101 was purchased from Sigma (Sigma-Aldrich GmbH, Sternheim, Germany). The lauric arginate (LAE, C_20_ H_41_ N_4_ O_3_ Cl, ≥98% purity, contained 20% LAE ± 1 in propylene glycol) with a commercial name of CytoGuard LA 2X was obtained from A&B Ingredients (Farfield, NJ). 4-(2-Hydroxyethyl)-1-piperazineethanesulfonic acid (HEPES) was purchased from Sigma-Aldrich (Steinheim, Germany). Free radical 2,2-diphenyl-1-picrylhydrazyl (DPPH), gallic acid (GA), and 2,4,6-tripyridyl-S-triazine (TPTZ) were supplied from Sigma-Aldrich (St. Louis, MO). Beet juice extract was purchased from Nature’s Bounty (Winnipeg, Manitoba, Canada). All other reagents used were of analytical grade and used without further purification.

### Synthesis of Grafted MCC Conjugate

To modify the surface of MCC, 3.0 g of MCC with an average particle size of 30 μm was homogeneously dispersed in 100 mL of deionized water and stirred for 30 min. Then, HEPES (0.5 g) was introduced into the MCC suspension and pH was adjusted to 8.5 by the addition of NaOH. This was followed by the sonication of the MCC suspension using an ultrasonic cleaning device (Bandelin 400, Berlin, Germany) at 10 kHz for 2 min. Next, the sonicated suspension was vigorously stirred at ambient temperature overnight. Afterward, the GA (0.3 g) was introduced into the MCC suspension (pH = 8.5) and stirred constantly by a magnetic stirrer. The reaction was done for 18 h at ambient temperature and under atmospheric pressure conditions. In the final step, LAE (0.3 g) was added to the MCC/GA suspension. This was continuously stirred for a further 18 h under ambient conditions. The product was then centrifuged (Eppendorf centrifuge 5417R, Hamburg, Germany) at 1409 G-force for 5 min and washed four times with deionized water, followed by four more times with ethanol. The MCC/GA/LAE particles were oven-dried overnight at 40 °C and stored for further characterization.

### Characterization of Grafted MCC Conjugate

#### Fourier Transform Infrared (FTIR) Spectroscopy

The transmission infrared spectra of the pristine MCC, MCC/GA, and grafted MCC conjugate were identified with an FTIR spectrometer (Jasco FT/IR6200, Tokyo, Japan) to ensure the presence of the grafting process. The solid samples needed for the FTIR assay were obtained in the pellet form by blending 10 mg of each sample with 100 mg of dry KBr. Next, the samples were transferred to pellets to scan the spectral area at the wavenumber range of 400 and 4000 cm^–1^, whereby 50 scans were recorded at 1 cm^–1^ resolution.^20^

#### Solid-State ^13^C NMR Spectroscopy

To further confirm the grafting of the MCC backbone upon the introduction of GA and LAE, solid-state ^13^C NMR was employed using a Bruker spectrometer (AvanceIII 500, Bruker, Ettlingen, Germany). The device was linked to a 4 mm MAS (magic angle spinning) probe, where the frequencies of carbons and protons were 75.46 and 300.13 MHz, respectively. The external reference was glycine which was utilized aimed at the ^13^C spectra and in order to set the Hartmann–Hahn matching condition in the cross-polarization experiments. The spectrum of each sample was obtained with the ramp {^1^H} → {^13^C} cross-polarization (CP)/MAS pulse sequence using the proton decoupling upon acquisition. The recycling period of 10 s and a contact time of 3 ms during CP were adjusted for all experiments. The SPINAL64 (small phase incremental alternation with 64 steps) sequence was employed for heteronuclear decoupling upon acquisition with a proton field H^1^H satisfying ω^1^H/2π = γHH^1^H = 62 kHz. The spinning rate for all samples was 10 kHz.^5^

#### X-ray Photoelectron Spectroscopy (XPS)

The XPS assay was also conducted to further verify the grafting modification of MCC. The experiments were conducted through a Kratos Axis spectrometer (Ultra Kratos Analytical, Manchester, U.K.) *via* a monochromatic Al Kα source and a 180° hemispherical electron energy analyzer, working at a pass energy of 65 eV. The step size was 0.1 eV with a dwell time of 1000 ms. The analyzed zone was adjusted at a region of 300 × 700 μm^2^. A Shirley baseline was employed aimed at the subtraction of the background, and Gaussian/Lorentzian (70/30) peaks were applied for spectral decomposition. Each spectrum was analyzed through Vision software supplied by Kratos (Vision 2.2.2, Ultra Kratos Analytical, Manchester, U.K.).

#### X-ray Diffraction (XRD)

The XRD diffractogram was obtained by an X-ray diffractometer (Shimadzu XRD 7000, Tokyo, Japan) with Cu Kα irradiation. The samples were exposed to the X-ray beam at 2θ angles ranging from 2 to 60° running at 45 kV and 40 mA, employing Cu Kα radiation (λ = 1.541 Å) at a rate of 2° min^–1^. To evaluate the relative crystallinity degree (RCD), the total curve area (*I*_t_) and the area under the peaks (*I*_p_) were determined using the software offered by Shimadzu, and RCD was then calculated according to eq 1(21)
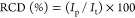
1

#### Water Contact Angle

The contact angle (CA) was obtained by an OCA 20 contact angle meter (Dataphysics Instruments GmbH, Filderstadt, Germany) using the sessile drop approach in natural light. A uniform thin film was fabricated using a KW-4A spin-coater (CHEMAT Technology Northridge, CA). This was achieved by spin coating of 2.0 wt % pristine and modified MCC suspensions (in toluene) onto silicon wafers at a shear rate of 210 s^–1^ (5690 G-force) for 1 min. This was then followed by heat treatment at 90 °C overnight. The resulting films were sectioned 4 × 6 cm^2^ and placed on a horizontal movable stage. A drop (5 μL) of deionized water with a syringe (10 μL, Hamilton, Switzerland) was deposited centrally on the surface of the films. The data were analyzed by the software offered by the Dataphysics Instruments.^22^

#### Scavenging Activity on DPPH Free Radicals

The stock DPPH solution was obtained by introducing 5.0 mg of DPPH in methanol (100 mL). The aqueous suspensions/solutions of pristine MCC, GA, LAE, and grafted MCC conjugate were individually prepared by dispersing 50.0 mg of each sample in 100 mL of deionized water and stirred for 60 min. Then, the DPPH solution (2 × 10^–4^ M, 100 μL) was blended with these aqueous suspensions/solutions (100 μL). The resulting mixtures were shaken vigorously and incubated at ambient temperature in the dark for 1 h. Next, the reactants were centrifuged (Eppendorf centrifuge 5417R, Hamburg, Germany) at 4000 G-force for 5 min. The absorbance was then measured at 517 nm using a spectrophotometer (UVIDEC-50, JASCO Corporation, Tokyo, Japan). The scavenging effect of DPPH radical was obtained as follows
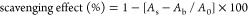
2where *A*_0_ is the absorbance of the control (using deionized water instead of the sample), *A*_s_ is the absorbance of the samples mixed with reaction solution, and *A*_b_ is the absorbance of the sample under the same condition as *A*_s_, but where ethanol was used instead of the ethanol solution of DPPH.

#### Total Antioxidant Capacity

The ferric reducing antioxidant potential was used to determine the total antioxidant capacity. The ferric reducing antioxidant potential working solution was provided by mixing 100.0 mL of sodium acetic buffer (0.3 M, pH 3.6), 10.0 mL of TPTZ (10 mM, dissolved in 40 mM HCl), and 10.0 mL of FeCl_3_ solution (20 mM).^23^ The mixture containing 100 μL of sample and 200 μL of the potential ferric reducing antioxidant solution was incubated at room temperature for 20 min, and the *A*_b_ and *A*_s_ values were detected at 593 nm.
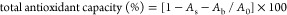
3

where, once again, *A*_0_ is the absorbance of the control (using deionized water instead of the sample), *A*_s_ is the absorbance of the samples mixed with a working solution, and *A*_b_ is the absorbance of the sample under the same condition as *A*_s_, but with ethanol used instead of the working solution.

#### Antimicrobial Properties

Three bacteria cocktails containing identical populations of five trial strains/serovars were utilized in the antimicrobial assessment, including (1) *Salmonella enterica* cocktail: *Salmonella montevideo*, *Salmonella gaminara*, *Salmonella agona*, S. Michigan, and S. Saint Paul; (2) *Escherichia coli* O157:H7 cocktail: H1730, K3995, F4546, 658, and 932; and (3) *Listeria monocytogenes* cocktail: LM1, LM2, 310, Scott A, and V7. Tryptic soy broth (TSB) was employed for *S. enterica* and *E. coli* O157:H7, and Tryptic soy broth supplemented with yeast extract (TSBYE) was applied for the growth of L. monocytogenes.^24^

A disk diffusion technique was applied to assess the antimicrobial features of the samples. First, the MCC film variants were fabricated by the spin coating method (see the Water Contact Angle section). Then, the tryptic soy agar (TSA) or TSA supplemented with yeast extract (TSAYE, for *L. monocytogenes*) plates were spread with 100 mL of culture containing 10^5^ CFU mL^–1^ of bacteria cocktail. Two circular disks of each film specimen (10 mm) were transformed into each plate. After incubation for 24 and 48 h at 32 °C (*L. monocytogenes*) or 37 °C (*E. coli* O157:H7 and *S. enterica*), the diameter (mm) of inhibition zones was determined using a ruler. The mean values of inhibition zone diameters from the two films, with two disks each (*n* = 8), are reported.

### Preparation of Soy Protein-Based Pickering Emulsion

The SPI aqueous dispersion was made by dispersing SPI powder (25.0 g) into part of the citrate phosphate buffer (pH 5.6, 60 mL), with the rest of the water being used for the grafted MCC conjugate. Next, salt (0.1 g) and beet juice extract (0.3% v/v) were introduced into the SPI-based dispersion. This was followed by gentle stirring of the SPI-based dispersion at 40 °C for 60 min, using a magnetic heater stirrer. At the same time, 10% (v/v) sunflower oil was incorporated into the SPI-based dispersion with a burette. The obtained emulsions were stirred by an Ultra-Turrax at a speed of 210 s^–1^ (5690 G-force) for 5 min. Separately, a stock suspension of grafted MCC conjugate was made by dispersing a weighed amount (70 wt %) of the grafted MCC powder into the same buffer (pH 5.6, 40 mL). This was mixed with a high-speed rotor-stator device (Ultra-Turrax T25D IKA, Germany) at a shear rate of 400 s^–1^ (20664 G-force) for 10 min at an ambient temperature. This grafted MCC suspension was gently stirred overnight at room temperature. The pH of the suspension was then adjusted back to 5.6.

An O/W emulsion was prepared by blending sunflower oil 10 and 90 wt % aqueous SPI-based dispersions (25.0 wt % SPI, pH 5.6) using a high-speed blender (Ultra-Turrax T25D IKA, Germany) for 5 min. This coarse emulsion was homogenized by a two-stage high-pressure Microfluidizer processor (M110-PS, Microfluidics international Corp., Newton, MA) with 1800 psi at the first stage and 700 psi at the second stage. The full-fat stabilized emulsion, regarded as control hereafter (10 wt % sunflower oil, 25.0 wt % SPI, pH 5.6), was employed for comparison with reduced-fat emulsions. Hence, 20, 40, 60, and 80% reduced-fat emulsions were prepared with grafted MCC stock suspension (70 wt % grafted MCC, pH 5.6) to obtain the Pickering emulsions with the following compositions: 8 wt % sunflower oil and 1.4 wt % grafted MCC (SGM1), 6 wt % sunflower oil and 2.8 wt % grafted MCC (SGM2), 4 wt % sunflower oil and 4.2 wt % grafted MCC (SGM3), and 2 wt % sunflower oil and 5.6 wt % grafted MCC (SGM4).

### Characterization of Soy Protein-Based Ink

#### Rheological Experiment

The rheological behavior of ink samples was characterized by an AR 2000ex rheometer (TA Instruments, New Castle, DE) using a parallel-plate geometry (diameter of 40 mm, gap size of 1 mm). The oscillatory strain sweep (0.1–100%, frequency = 1 Hz) was performed to attain the limit of the linear viscoelastic region (LVR). Apart from this, the frequency sweep test (0.1–100 Hz) was accomplished within the LVR (γ = 1%). All measurements were performed at 25 °C.^19,25^

To evaluate the steady rheological properties, the shear stress was measured as a function of increasing shear rate (γ̇) from 0.1 to 100 s^–1^. The best constituent rheological equation was selected *via* statistical analysis, and the rheological variables were measured using fits to the optimum model.^26^ Hence, the consistency index, flow behavior index, and yield stress values were obtained by fitting the Herschel–Bulkley model to the data (eq 4).
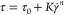
4where τ is the shear stress (Pa), τ_0_ is the yield stress (Pa), *K* is the consistency index (Pa s*^n^*), γ̇ is the shear rate (s^–1^), and *n* is the flow behavior index.

#### Creep and Creep-Recovery Test

The creep and creep-recovery measurements were performed to evaluate the compliance level during the creep and recovery stages *via* an AR 2000ex rheometer (TA Instruments, New Castle, DE). First, a stress sweep (1 Hz, 0.1–10 Pa) was accomplished (data not shown) to evaluate the oscillatory yield stress (*G*′(τ) = *G*″(τ)), and then the obtained values were considered as being about 50% of the yield stress.^5,19^ The inks were moved to a parallel-plate geometry with a diameter of 40 mm and a gap size of 1 mm, maintained at 25 °C. The creep measurement included the prompt application of a constant shear stress within the LVR area, lasting from 0 to 500 s while evaluating the sample deformation during these time intervals. Regarding the recovery phase, the applied stress was rapidly removed (τ_applied_ = 0.0 Pa) and the recovery values were recorded for an additional 500 s at the same temperature as that in the creep phase.^19^ The calculated strain and the recovery are considered to be the creep compliance and creep-recovery compliance (*J*) (eq 5). The creep-recovery percentages of inks were then obtained according to eq 6
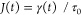
5
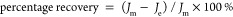
6where *J*(*t*) (Pa^–1^) is creep compliance at time *t*, γ is the measured strain, τ_0_ is the constant applied shear stress, *J*_m_ (Pa^–1^) is the maximum creep, and *J*_e_ (Pa^–1^) is the equilibrium creep compliance after recovery.

#### Three Interval Thixotropy Test (3ITT)

3ITT involved a three-step shear rate test, where the first one comprised a steady shear rate to recognize the ink reference stage without interrupting the microstructure. This was accomplished with a fixed shear rate of 1 s^–1^ for 400 s. In the second interval, a steady shear rate of 80 s^–1^ was applied for 200 s and used to terminate the microstructure of the ink. The third interval included a similar assessment condition as the first interval, allowing the partial (or full) recovery of the original structure of the Pickering emulsion gels^19^ (speed and degree of recovery).

### Printing Process

The 3D printing process of prepared soy protein-based inks was conducted using an extrusion-based system (nScrypt-3D-450, nScrypt, Orlando, FL), coupled with a syringe pump (PHD Ultra; Harvard Apparatus Holliston, MA). With the use of computer-aided design software (AutoCAD; Autodesk, Inc., San Rafael, CA), a special circle and butterfly-shaped objects were modeled and converted to an STL file. The print paths were provided through the creation of the G-code files to control XYZ direction instruction for the printer, developed by the open-source CAM software Slic3r (slic3r.org, consulted on December 2020) from the STL file. The printable soy protein-based inks were poured into a stainless-steel cartridge (10 mL) and stirred with a Vortex mixer (Fisher Scientific, Ontario, Canada) for 10 min, thus removing any air bubbles from the ink. The layer height was set at 1 mm, indicating that the nozzle tip was elevated by this value upon completion of the fabrication of each layer and before commencing the construction of the next layer. The process was continued until the suitable 3D architecture was printed in each case. The number of deposited layers was 8, and the width of the tip was 1 mm (Supporting Information, Table S1).

### Characterization of 3D-Printed Objects

#### Printing Performance Measurement

Each 3D-printed object was transferred into a specific chamber 20 × 20 × 20 cm^3^ to be photographed using a digital camera (α 7M3 E-Mount, Full-Frame Mirrorless, 24.2 MP, Sony, Tokyo, Japan). The printing performance of 3D-printed architectures was accomplished by determining the line width and layer number through a digital caliper (Mitutoyo, Absolute Digimatic, Tokyo, Japan).^6^

#### Temporal Dominance of Sensations (TDS)

Ten assessors (five females and five males, aged: 20–35 years) were selected to evaluate variances in the dynamic sensory profile of 3D-printed meat analogue through the TDS method.^27^ In this regard, six selected attributes were listed as being firmness, juiciness, oiliness, graininess, fibrousness, and chewiness (Supporting Information Table S2). The TDS evaluation was performed over three sessions to run three replicates. The 3D-printed objects 5 × 5 cm^2^ were provided to the assessors by the randomized complete block design in a monadic order. The panelists were then presented with a list of six attributes on the computer screen, each associated with an unstructured scale, ranked from weak to strong. FIZZ software (Version 1.9, Biosystems, Counternon, France) was utilized to obtain the TDS plots. The TDS graphs were smoothed by MATLAB software (R2016a, MathWorks, Inc., Natick, Ma) to generate TDS curves.^28^

### Statistical Analysis

All instrumental experiments were carried out in triplicate with the mean and standard deviation of the data calculated and reported. Analysis of variance (ANOVA) was utilized for the determination of the main effects and to examine the independence or the interactions between various factors on the instrumental and sensory data. Duncan’s multiple range test was applied to separate means of data when significant differences (*P* < 0.05) were observed.

## Results and Discussion

### Characterizations of Grafted MCC Conjugate

**Figure 1:**
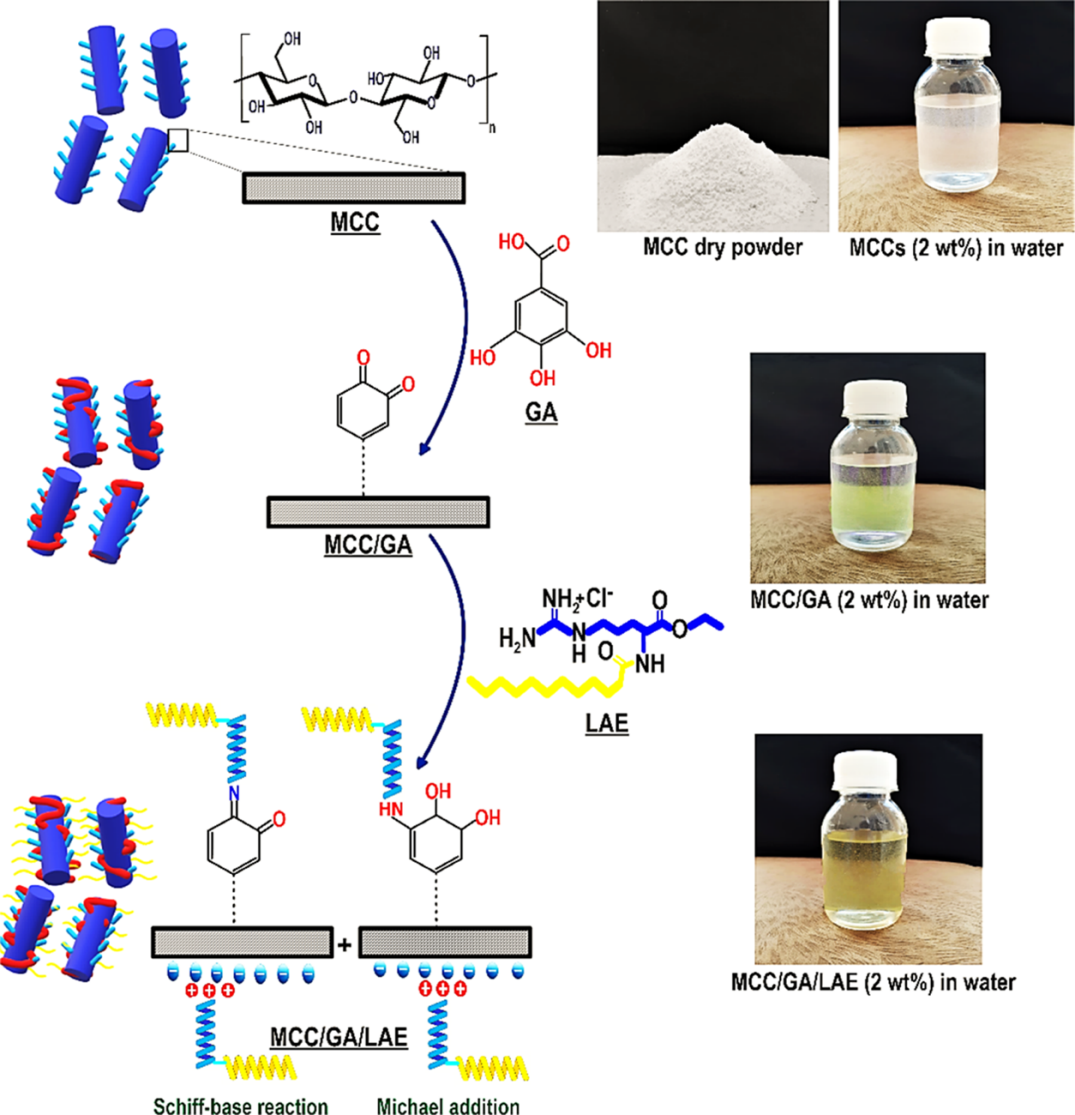
(Left) Products produced from the LAE reacting with a Schiff-base reaction and Michael addition. The dotted line signifies GA grafting onto the MCC *via* covalent and/or strong intermolecular interactions, as reported for other substrates (10). (Right) Photographs displaying the presence of modified MCCs in different phases regarding the one-pot water-based grafting modification.

The pristine MCC powder was purchased as a freeze-dried powder, which was simply redispersed in water with the shearing treatment (Figure 1). The introduction of GA into the MCC dispersion upon the sonication process resulted in the coated MCC (MCC/GA) that preserved the colloidal stability of MCC albeit with a somewhat yellow discoloration (Figure 1). On the other hand, the development of the grafted conjugate in the presence of LAE caused an increase in turbidity. This could imply an increase in the amphiphilicity of the system upon the grafting process and also the formation of complex coacervates between MCC and LAE.^10^ It was stated that the interaction of LAE with other negatively charged molecules leads to the formation of complex coacervates.^29^ This finally decreases the efficiency of LAE and therefore reduces the minimum inhibitory concentration of the LAE in certain food and pharmaceutical applications. Hence, there is a need to decrease the coacervate development and therefore enhance the efficiency of LAE. In the current study, we used grafting GA onto the MCC backbone to reduce the possible formation of large aggregates that leads to low turbidity and large particle size. The possible mechanism for the surface grafting of MCC as affected by introducing GA and LAE is schematically shown in Figure 1. It has been stated that interaction among biopolymers and oxidized polyphenols leads to the development of new inter- or intermolecular interactions including covalent and hydrogen linkages, metal chelation, and π effects.^30^ In the first phase, GA is reacted with MCC at pH 8.5 since it is recognized that under such circumstances, polyphenol oxidation and oligomerization can proceed. This is especially the case when dissolved oxygen is present. The process leads to the development of a high-molecular-weight compound with reduced solubility. Apparently, the solubility reduction of GA or its intrinsic affinity to MCC results in its grafting on the surface of MCC.^31^ In this situation, quinones are produced and, in the subsequent reaction phase, react by −NH_2_ of LAE through the Schiff-base and/or Michael-type addition reactions (Figure 1).

### FTIR Measurement

**Figure 2:**
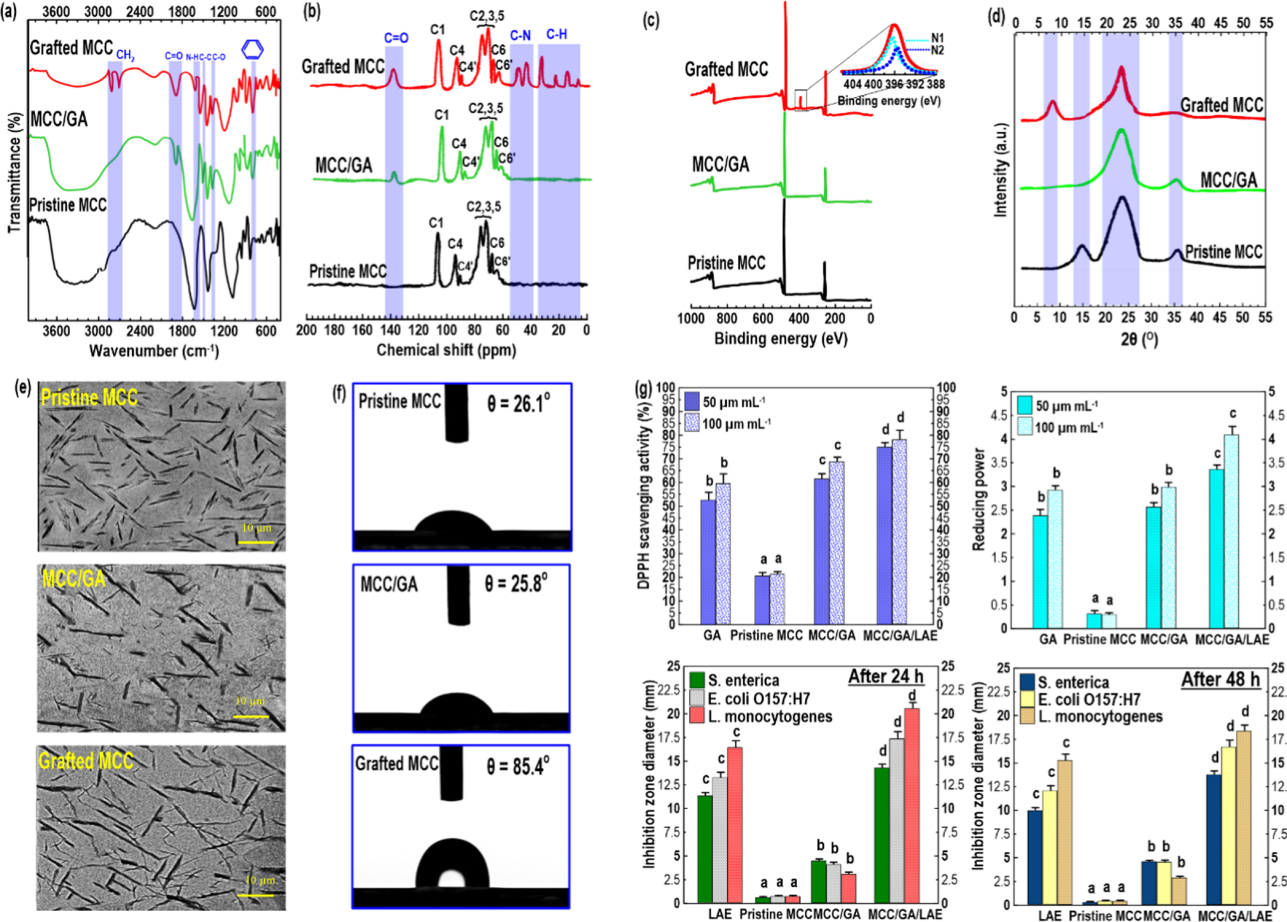
Characterization of grafted MCC conjugate: (a) FTIR, (b) ^13^C NMR, (c) XPS, (d) XRD, (e) transmission electron microscopy (TEM), (f) water contact angle, and (g) bioactive properties. In the case of bioactive features, the means inside each column with various letters (a–d) are significantly different (*P* < 0.05) according to Duncan’s test.

Figure 2a shows the FTIR spectra of unmodified MCC, MCC/GA, and MCC/GA/LAE products. A characteristic peak of cellulose I, centered from 2500 to 3750 and 700 to 1800 cm^–1^, was detected for pristine MCC. Moreover, the stretching of the hydroxyl group appeared around 3380 cm^–1^. There are also some representative bands at 2953 cm^–1^ (assigned to C–H stretching), 1640 cm^–1^ (related to asymmetric stretching of a carboxyl group), 1430 cm^–1^ (associated with methylene symmetrical bending), 1080 cm^–1^ (allocated to cellulose C–O–C bridge), and a peak around 880 cm^–1^ that corresponds to β-linked glucose moieties.^5^ The FTIR spectrum of MCC/GA evidently shows the emergence of a new typical carbonyl stretching vibration around 1869 cm^–1^. Moreover, the band induced by −COOH groups shifted to the higher wavenumbers (1687 cm^–1^). However, the main difference between the pristine MCC and MCC/GA was the appearance of a band associated with C–O stretching, occurring around 1340 cm^–1^ for GA-coated MCC.^32^ In addition, also a new peak appeared around 791 cm^–1^, resulting from the distortion of the vibrations of MCC in the benzene rings, and a further band at about 1510 cm^–1^ caused by the stretching vibration of the C–C aromatic ring. These changes are suggestive of the interactions between MCC and GA. A relevant FTIR pattern was also stated by Hu et al.^31^ The FTIR spectrum of the grafted MCC/GA/LAE conjugate exhibiting the magnitude of the stretching band of hydroxyl groups (−OH) was wider and less pronounced (Figure 2a). This is likely because of the higher consumption of −OH groups of MCC, resulting from the grafting process. The grafted MCC conjugate also displayed the presence of a band around 1340 cm^–1^ that was thought to be the result of C–O stretching. At the same time, the asymmetrical/symmetrical stretching of methylene results in bands at 2705 and 2810 cm^–1^. This suggests the likely grafting of LAE to the MCC surface. Furthermore, the typical secondary N–H bending around 1490 and 1603 cm^–1^ confirmed the occurrence of Michael addition.

### ^13^C NMR Spectroscopy

The grafting treatment of MCC was qualitatively further verified by ^13^C NMR spectroscopy (Figure 2b). A characteristic band of cellulose I (*i.e*., C1: 110.9 ppm, C4: 98.1 ppm, C4′: 95.3 ppm, group C2–C3–C5: 70.2–80.1 ppm, C6: 69.1 ppm, and C6′: 64.9 ppm) was detected for pristine MCC.^5^ After GA grafting onto MCC, no obvious new peaks emerged in the NMR spectrum compared to pristine MCC. However, a relatively wide band appeared at about 145.3 ppm, which associates with the resonance of the carbonyl ester (C=O). This result was in accordance with the carbonyl stretching vibration around 1869 cm^–1^ detected by FTIR assay. In contrast, the changes of the NMR spectrum of grafted MCC/GA/LAE conjugate were more marked compared to GA-coated MCC. As the grafting reaction progressed, new strong resonances emerged in the NMR spectrum, as seen by the appearance of distinguished signals at 8.2, 13.2, 24.3, 34.3, 41.8, and 45.5 ppm. These changes could be caused by the terminal methyl carbons, *i.e*., the secondary carbon (−CH_2_−) and the primary carbon groups (−CH_3_). The obtained data further supported the effective grafting process of GA and LAE onto the MCC backbone. Moreover, the crystallinity degree measured by NMR demonstrated that only a small alteration in the crystallinity index of MCC (about 85%) occurred upon the grafting treatment (∼86%) (Supporting Information, Section S.1.1).

### XPS Measurement

The XPS patterns of the pristine MCC, GA-coated MCC, and grafted MCC conjugate are compared in Figure 2c, providing yet further support for the indicated grafting mechanism. As expected, the XPS spectrum obtained from the pristine MCC identified oxygen and carbon. After the grafting treatment, a nitrogen peak also emerged on the XPS pattern of the grafted MCC conjugate. This latter peak results from LAE. The resolving of the nitrogen peak (396.3 eV) led to the identification of binary compounds, N1 (395.5 eV) and N2 (397.1 eV). They were likely induced by aromatic C=N and aromatic C–N, respectively.^33^ The presence of both aromatic C=N and C–N bands denotes the formation of both Schiff-base reaction and Michael addition between MCC/GA and LAE.^33^ Additionally, the peak intensity at 266.3 and 267.1 eV was increased, which could also be related to the aromatic C=N and aromatic C–N, respectively. This then also implies the successful LAE grafting on the GA-coated MCC backbone. Per the XPS patterns, some general interpretations regarding atomic ratios could be made. The theoretical proportion of O to C in pure cellulose is known to be 0.83.^34^ In the current study, the obtained O/C ratios were 0.79, 0.91, and 0.42 for pristine MCC, GA-coated MCC, and grafted MCC conjugate, respectively. The obtained data for the grafted MCC conjugate was not surprising due to the presence of R–NH_2_ chains in the LAE structure with no oxygen, where the O/C proportion reduced significantly. The discrepancy in O/C proportion regarding MCC and GA-coated MCC might also be ascribed to slight contamination.^31^ It is worth noting that the GA layer on the GA-coated MCC was relatively thin since if the XPS beam only interacted with GA (with a certain penetration depth of ∼10 nm), a much lower O/C ratio of 0.73 (O/C ratio for neat GA) would be anticipated.

### XRD Pattern

The diffractogram of pristine MCC exhibited a crystalline structure with the dominance of cellulose type I in the MCC.^5^ In this case, there are some strong reflections around 2θ = 14.0° (*d*_001_ = 5.4 Å), 2θ = 22.9° (*d*_001_ = 4.8 Å), and 2θ = 34.9° (*d*_001_ = 4.0 Å) (Figure 2d), whose RCD was measured to be 78%. This crystallinity index was slightly lower than the crystallinity degree of about 84% obtained from the NMR experiment. The diffractogram of GA-coated MCC presented that the GA grafting onto the MCC resulted in the fading of the pronounced peak located around 2θ = 14.0°, which signified the interaction of GA with MCC in the interhelical backbone. The RCD of this sample was also dropped to 71%. The obtained result signifies a reduction in the magnitude of the crystalline reflections together with a decline in the magnitude of the pronounced diffraction peak of MCC. Moreover, the characteristic peak of MCC shifted from 2θ = 22.9° to 2θ = 22.2°, which indicates that the gallery spacing from *d*_001_ = 4.8 Å (2θ = 22.9°) increased to *d*_001_ = 4.9 Å (22.2°). It has been reported that there is a slight change in the spatial structure and unfolding of the cellulose upon the interaction of functional groups of GA with MCC.^10^ As Figure 2d reveals, the intensity of diffraction peaks decreased more upon the development of a grafted MCC/GA/LAE conjugate, whose RCD dropped down to 58%. The XRD diffractogram showed that the typical MCC peak (2θ = 22.9°) altered to a more diffused peak, showing a reduction in the crystallinity. Furthermore, the diffractogram of the MCC/GA/LAE conjugate displayed the appearance of a new reflection around 2θ = 8° (*d*_001_ = 6.1 Å). This could indicate that a new crystalline phase was developed in the amorphous area of cellulose through the grafting process.

### Morphological Evaluation

Figure 2e shows the transmission electron microscopy (TEM) photograph of unmodified MCC, MCC/GA, and grafted MCC/GA/LAE products (For an interpretation of the reference for the preparation of samples for TEM analysis, the reader is referred to Supporting Information Sections S2). In the case of unmodified MCC, the particles are rod-shaped and are well separated from each other, with no noticeable agglomeration. Compared to pristine MCC, the size of particles in the GA-coated MCC and grafted MCC conjugate had increased, but nonetheless, they preserved their rod-shaped morphology and remained as well-dispersed individual particles, without any significant levels of agglomeration. Furthermore, there was an enhancement of the contrast dark coating on the surface of modified MCC. It has been stated that an increased contrast of dark coating in the modified MCC is probably because of the existence of aromatic groups in the phenolic compounds.^31^

### Contact Angle

The model MCC thin film was made by the spin coating approach, and a sessile drop water contact angle experiment was performed. The pristine MCC film presented a high hydrophilicity character, showing a low water contact angle of θ = 26.1° (Figure 2f). In this case, the water droplet was completely absorbed into the MCC film after 1 min. The MCC backbone is quite wettable due to the presence of a large number of hydrophilic groups.^35^ The contact angle measurement also exposed that the grafting of GA onto the MCC backbone did not alter the hydrophobicity of the MCC matrix (θ = 25.8°), specifying the lack of development of a hydrophobic surface/matrix in the GA-coated MCC. As Figure 2f shows, a huge enhancement in the contact angle by a value of 59.3° was detected after the formation of grafted MCC conjugate (θ = 85.4°). This could be related to the progress of intermolecular interactions between MCC, GA, and LAE due to the grafting reaction. This provides fewer hydrophilic sites on the surface of films, but with more matrix rigidity.^31^

### Antioxidant Activity and Reducing Power

The antioxidant behavior of a compound is related to several mechanisms, including breaking the radical chains, hampering metal-catalyzed initiation reaction, and inhibition of the introduction of initiating radicals.^36^ To determine the antioxidant capacity, different in vitro chemical-based experiments, including assays of radical scavenging activity on DPPH free radicals and ferric reducing antioxidant power, were performed. Among all evaluated samples, the unmodified MCC presented the lowest DPPH scavenging effect (Figure 2g). The scavenging activity of GA alone on the DPPH free radicals was also measured. This illustrated that the scavenging effect was appreciably higher than the pristine MCC. GA is considered a strong apoptosis-inducing agent and an effective antioxidant agent.^10,37^ Thus, the introduction of GA to MCC could reasonably induce an efficient antioxidant property with a promising therapeutic effect. Compared to the free GA compound, the GA-coated MCC and grafted MCC/GA/LAE conjugate provided a stronger quenching of DPPH radicals in a dose-dependent manner (*P* < 0.05). It was reported that polyphenol oxidation and oligomerization are developed after the grafting of GA onto the MCC backbone.^10^ This enlarges the size of the conjugated compound of GA, contributing to an increase in the electron-donating groups. Thus, the GA-coated MCC and MCC/GA/LAE conjugate have more biological stability than the free GA. We assume then that these products, with their swollen supramolecular structure, could capture the free radicals more successfully compared to the small GA molecules.

Another important parameter to evaluate the antioxidant activity of a compound is the ferric reducing antioxidant power.^10^ The reducing power activity of different samples is shown in Figure 2g. The reducing power of all samples, except unmodified MCC, was increased in a dose-dependent manner. The unmodified MCC showed a weak reducing antioxidant power. The GA-coated MCC, by contrast, offered stronger reducing power compared to unmodified MCC (*P* < 0.05) and was similar to the neat GA (*P* > 0.05). This is expected since GA is established for its excellent antioxidant feature *via* the active hydrogen-donating groups.^10,37^ The reducing power activity had enhanced even further for the grafted MCC/GA/LAE conjugate (Figure 2g). The obtained data suggest that the interaction of MCC by both GA and LAE improved the antioxidant behavior of unmodified MCC. In this regard, high-molecular-weight species are developed upon the grafting process that provided a biological stable compound, strongly mopping up the free radicals more efficiently compared to free GA molecules on their own.^10^ Thus, the idea to reinforce the antioxidant activity by grafting GA and LAE onto the MCC backbone seems a sensible one.

### Antimicrobial Activity

The antimicrobial activities of free LAE compound, pristine MCC, GA-coated MCC, and grafted MCC/GA/LAE conjugate were assessed by the disk diffusion experiment upon different incubation conditions (Figure 2g). The pristine MCC did not show an inhibitory effect against any of the evaluated microorganisms, where some bacteria growth was detected under the MCC films’ disks. In contrast, a slightly larger inhibition zone diameter was observed for the disks of GA-coated MCC (*P* < 0.05), albeit the difference was not significant after 24 and 48 h (*P* > 0.05). A great inhibition zone diameter was also detected for disks of grafted MCC conjugate, with the differences now being significant after 24 and 48 h (*P* < 0.05). This demonstrated a continuous inhibitory effect of film disks of the grafted MCC/GA/LAE conjugate. The antimicrobial effect of the LAE component^29^ in the grafted system was also in accordance with the inhibitory effect of LAE alone, as conducted in the present study (Figure 2g). The presence of a positive charge on the protonated guanidine group of LAE can disrupt the cell membranes of microorganisms without triggering the cell lysis. However, this mechanism might also act toward various intracellular bacterial pathogens, which can also prove lethal to the bacteria.^24,29^ In the current work, the antimicrobial MCC offered a great inhibitory effect against *L. monocytogenes* compared to *S. enterica* and *E. coli* O157:H7, which was also reported by Pattanayaiying, Aran, and Cutter.^38^ Following 24 h of incubation, the film disks of grafted MCC/GA/LAE conjugate presented meaningfully higher inhibition zones to inhibit all three types of bacteria studied here when these were compared to GA-coated MCC film disks (*P* < 0.05). This shows a promising efficiency of grafted MCC/GA/LAE conjugate to enhance product safety in possible industrial applications. Compared to the grafted MCC/GA/LAE conjugate film disks, the GA-coated MCC film disks showed significantly lower inhibition zones (*P* < 0.05). The obtained data might have resulted from the lower inhibitory effect of GA in comparison to LAE.

### Characterization of Soy Protein-Based Pickering Emulsions

#### Flow Curve of Soy Protein-Based Pickering Emulsions

**Figure 3:**
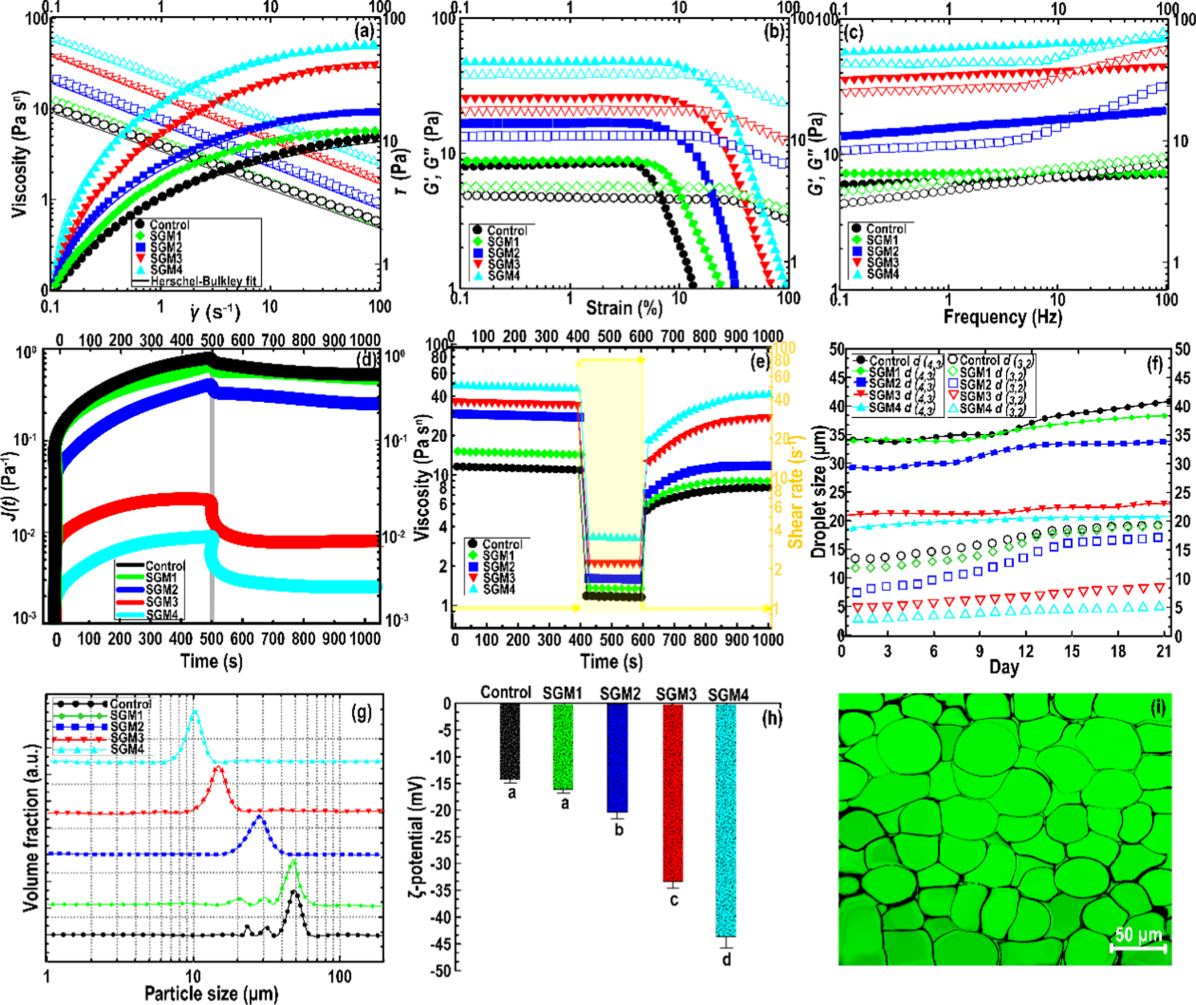
(a) Flow curve of soy protein-based emulsions. (b) Amplitude sweep and (c) frequency sweep of soy protein-based emulsions, where *G*′ is denoted by solid symbols and *G*″ is denoted by open symbols. (d) Creep and creep-recovery curves and (e) 3-ITT of prepared Pickering emulsion variants. (f) Droplet size, (g) particle size distribution, and (h) ζ-potential (For more insight into these tests, see Supporting Information Section S3) of different soy protein-based inks. (i) Confocal laser scanning microscopy (CLSM) image of control emulsion (For more insight into the CLSM test investigation, see Supporting Information Section S4).

To be appropriate for 3D printing, a biopolymeric ink must simply be squeezed out during the process, with reduced viscous flow forces inside the nozzle at the time of its application.^3,5,10^ However, the ink must also become viscous enough to keep the 3D-printed shape once the printing process is completed. Therefore, it is obvious that such an ink must exhibit a significant degree of pseudoplasticity. Figure 3a presents the flow curves of soy-based Pickering emulsions. Each plot was fitted to the Herschel–Bulkley equation, showing a high correlation coefficient above 0.97 (Supporting Information Table S3). As given in Table S3, the oil replacement by MCC/GA/LAE conjugate (micro-biosurfactant) led to a lower flow behavior index, suggesting that the Pickering emulsions have become more shear thinning. Generally, the inks formulated with the higher levels of grafted MCC conjugate, *i.e*., SGM3 and SGM4, provided lower flow behavior indices, with a clear pseudoplastic behavior (0.455 < *n* < 0.542) (Supporting Information Table S3). The presence of the less aggregated droplets/particles and more efficient dispersed particles in the inks including higher contents of grafted MCC/GA/LAE conjugate could be that the aggregates become less strong and break more easily at the lower shear forces, thus providing rapid shear thinning.^5,19^

The viscosity, which corresponds to the consistency index of an emulsion, offered an appreciable increase when the grafted MCC conjugate was replaced by oil in the range of 4.2–5.6 wt%, *i.e*., SGM3 and SGM4 (Figure 3a). The SGM4 ink presented the highest viscosity (at a shear rate of 10 s^–1^) with an initial value of 41.7 Pa s, followed by SGM3 and SGM2 with a viscosity (at a shear rate of 10 s^–1^) of 38.5 and 34.5 Pa s, respectively (Supporting Information Table S3). The greater viscosity value with grafted micro-biosurfactant is likely because of the development of intermolecular interaction among the hydrophobic regions of LAE and oil on the one hand and more polar cellulose chains with the hydrophilic domains of soy protein on the other. Moreover, the higher solid volume fraction in the system upon the addition of grafted MCC may increase the particle–particle interactions, leading to the formation of weak but large clusters and decreased mobility that in turn causes an increase in viscosity.^5^

The yield stress difference is considered an important property of printable inks. It is associated with the reinforcement of ink printing performance in addition to the development of well-defined 3D structures.^3^ There was an increase in the yield of soy protein-based ink upon the addition of grafted micro-biosurfactant (Supporting Information Table S3). Compared to control ink, replacement of the lowest level of grafted MCC conjugate did not significantly alter the yield stress (*P* > 0.05), while at higher ratios, it provided a noticeable increase in this parameter (*P* < 0.05). The introduction of GA and LAE to the MCC backbone presents the possibility for linking a diverse range of polymeric chains, *via* the development of phenolic dimers/trimers and/or even polyphenol oxidation and oligomerization. The new linkages might result in greater molecular weight and longer chain lengths, requiring higher stress to make the ink flow. Thus, it was assumed that the high yield stress of the inks containing higher levels of grafted MCC conjugate was ascribed to the cross-linking junction zones obtained by the oxidation of GA followed by *in situ* reacting LAE *via* Schiff-base formation and/or Michael-type addition. The obtained results are imperative since the desired range of yield stress prevents the deformation of the extruded layers and the collapse of the 3D-printed structures.

#### Strain Sweep of Soy Protein-Based Pickering Emulsions

The dynamic rheological behavior of inks was taken into consideration as it can offer valuable insights into the viscoelastic parameters of biopolymeric inks, which directly affects the printability and resolution of deposited layers.^3^ The results of the strain sweep test are depicted in Figure 3b. All emulsions presented a *G*′(γ) higher than *G*″(γ) for a wide range of strain amplitude, exhibiting the gel-like character of the Pickering emulsion gel. The *G*′ modulus shows a sign of the system rigidity; then, the inks with a greater *G*′ offer a more desirable structural strength and may aid to form a well-defined geometry upon the printing process. Compared to the control ink, the inks containing grafted MCC conjugate presented greater viscoelasticity throughout the entire period of the strain sweep. Besides, the *G*′(γ) values increased at higher ratios of grafted micro-biosurfactant. Moreover, the amphiphilic chains with several hydrophobic regions/groups along the backbone tend to become simultaneously adsorbed on the surface of two nearby droplets, thus resulting in the bridging flocculation of droplets. If droplets remain stable against coalescence, then the resulting network formed by these oil droplets exhibits strong viscoelastic behavior.^5,19^

Additionally, the linear viscoelastic region (LVR), where moduli remain independent of the amplitude of oscillations, was also evaluated to confirm that the experiment was carried out within LVR. The extent of LVR can reflect the strength of any formed network in the system. Stronger structured emulsion gels could remain within the LVR over a higher level of deformation compared to the weak gels.^39^ As Figure 3b depicts, the control emulsion possessed a short LVR extent, showing the lower critical strain (2.3%) in comparison with other samples, whereas the critical strain values of SGM1, SGM2, SGM3, and SGM4 inks were determined to be higher at 3.8 5.6, 8.2, and 9.6%, respectively. As a result, compared to the lower contents of the grafted MCC conjugates, the higher ratios of the grafted micro-biosurfactant offered a greater *G*′_LVR_ with a less restricted LVR, thus also a higher critical strain. This specifies that the reduced-fat ink variants behaved more like a solid with elastic-like features, signifying the development of more structured systems under nondestructive conditions.

The loss modulus (*G*″) is an index of the dynamic viscous character that signifies the dissipation of energy linked to the unrecoverable viscous loss. As illustrated in Figure 3b, all ink variants showed a constant *G*″(γ) trend within LVR, as expected. However, at higher amplitude sweeps (>7%), *G*″(γ) plots crossed over with those of *G*′(γ), indicating a shift from more elastic behavior to a more viscous liquid-like one. The crossover points of the reduced-fat inks were transitioned to the higher strain amplitudes compared to control the ink. This suggests the development of a stronger gel-like matrix with a more structured behavior.^5^ As an outcome, the enhancement of viscoelastic properties of the Pickering emulsion gels resulting from the addition of a high content of grafted micro-biosurfactant may better preserve their shapes upon printing and enhance the printability and also the geometrical retention of 3D architectures.

#### Frequency Sweep of Soy Protein-Based Pickering Emulsions

Figure 3c shows the viscoelastic moduli as a function of oscillatory frequency (ω) inside LVR (γ = 1%). Concerning the control and SGM1 inks, *G*′(ω) dominated over *G*″(ω) at low frequencies (<1 Hz), demonstrating the dominance of quasi-solid behavior. However, as the frequency proceeded, *G*″(ω) gradually approached more closely the value of *G*′(ω) until a crossover point (ω_c_), *i.e*., *G*′(ω) = *G*″(ω). On the other hand, the moduli of the SGM2, SGM3, and SGM4 inks all presented a relatively linear rise by increasing the frequency. In these cases, the intensity of *G*′(ω) was also considerably higher as the ratio of grafted micro-biosurfactant was increased, with the stronger gel-like feature. It is proposed that the development of GA-coated MCC contains the oxidation of phenolic group for producing *o-*quinones or *o-*semiquinones.^10,30^ The orthoquinone then *in situ* reacted with the amino groups of LAE through Schiff-base development and/or Michael-type addition, which improves the hydrophobicity contact angle for the surface of MCC. MCC particles with higher hydrophobicity tend to aggregate to form stronger networks. This most likely includes MCC particles adsorbed on the surface of droplets, hence incorporating droplets into the MCC gel network, as active fillers.

The reinforcing effects of the grafted MCC/GA/LAE conjugate were also reflected on the *G*″(ω) modulus. In this case, *G*″(ω) linearly increased as the micro-biosurfactant level was increased. This offers the strengthened structure of soy protein-based emulsion and a mechanically stable matrix, showing an elastic gel-like character. As Figure 3c depicts, the slope of *G*″(ω) for the reduced-fat inks was higher than *G*′(ω) at higher frequencies (>10 Hz), where the crossover point (ω_c_) was observed. Moreover, a shift in ω_c_ to higher frequencies was also noticed with increasing content of grafted micro-biosurfactant. The formation of viscoelastic Pickering emulsion systems offered by the grafted MCC conjugates could be a valuable way for the realization of the soy protein-based ink. This helps to keep the printed shape upon extrusion force during 3D printing and reinforces the mechanical strength and shape fidelity.

#### Creep-Recovery of Soy Protein-Based Pickering Emulsions

From Figure 3d, the creep compliance levels of SGM3 and SGM4 inks were lower than those of SGM1 and SGM2 samples. As the creep-recovery data presented, the SGM3 and SGM4 showed a maximum creep compliance, which are about 33-fold (*J*(t) = 0.026 Pa^–1^) and 96-fold (*J*(*t*) =0.009 Pa^–1^) lower than the control ink (*J*(*t*) = 0.86 Pa^–1^), respectively. Compared to SGM3 and SGM4, the SGM1 (*J*(t) = 0.75 Pa^–1^) and SGM2 (*J*(t) = 0.48 Pa^–1^) inks presented a relatively weaker structure, caused by a larger peak strain level (Figure 3d). Therefore, the greater ratios of grafted MCC conjugate could result in a greater improvement of the elastic component of the viscoelasticity. This might be explained by the existence of higher amounts of hydrophobic/hydrophilic regions in Pickering emulsion gels.^10^ In this situation, a strong interaction could be developed between comparable groups in the adjacent droplets/particles.^5^

The recovery area of the creep test can be considered as the amount of decline in the compound deformation upon the removal of the applied stress.^5,10^ A material with higher elasticity and a more gel-like matrix offers a greater relative recovery.^40^ The recovery phase data revealed that the relative recovery property of soy protein-based ink was enhanced with increasing level of the grafted MCC conjugate. In this regard, the recovery percentages of SGM3 (∼70%) and SGM4 (∼75%) inks were much higher than the control ink (∼45%), hence signifying an appreciable reinforcement of the recovery properties of the ink system. A poor relative recovery, by contrast, was noticed for SGM1 and SGM2 with a recovery percentage of around 45 and 50%, respectively. This indicated weaker elasticity and mechanically a less stable structure. The creep-recovery data point to the incorporation of a higher level of micro-biosurfactant that formed a strengthened ink structure. According to the creep-recovery test, a more reversible network matrix in the reduced-fat inks was induced after introducing the micro-biosurfactant, which affects the elastic or viscous behavior of the viscoelastic properties of the soy-based printable ink.

#### 3ITT of Soy Protein-Based Pickering Emulsions

The 3ITT measurement was used to evaluate if the Pickering emulsions are prone to rapid recovery upon being sheared at large deformations. As a criterion, when the peak viscosity of a material in the third interval recovers to at least 70% of its initial value, it has shown an ideal thixotropic behavior.^40^Figure 3e presents the changes in the viscosity of printable ink variants as a function of shear rate and time. Compared to control ink, the viscosity of reduced-fat inks, formulated by grafted MCC conjugates, presented greater values as may be expected. This signifies an improvement of the structural strength. Concerning the initial shearing interval, SGM3 and SGM4 inks displayed a considerably higher viscosity value compared to SGM1 and SGM2. The greater viscosity, attributed to a greater amount of micro-biosurfactant, can be ascribed to the more hydrophobic nature of MCC/GA/LAE conjugate, leading to stronger bonds holding particles together in the formed colloidal gel networks. Concerning the third stage, a lower viscosity was detected for all inks than that of the first stage. Regarding the 3ITT, the final viscosity transients expose the changes in the structural levels of the system once the shear rate is suddenly stepped up or down.^10^ Thus, a change in the viscosity of Pickering emulsion gel, after application of a stepwise shear rate, is related to its viscoelasticity and recovery compliance resulting from its structures.^5^ In the second interval (with a steady shear rate of 80 s^–1^), a marked reduction in viscosity was observed due to the breaking up of the high levels of interconnected structures in the Pickering emulsion gels (Figure 3e). Once shear was removed (*i.e*., the third interval), the reduced-fat inks showed a reversible restructuration toward their initial structure. As displayed in Figure 3e, the SGM3 and SGM4 inks presented an outstanding level of structural recovery, with initial viscosity recoveries were determined to be 76 and 84%, respectively. This follows the expected trend of structuring of the Pickering emulsion gel resulting from the micro-biosurfactant addition, thus allowing a higher resistance to the application of a rapid strain. In contrast, the lower extents of viscosity recoveries of SGM1 (38%) and SGM2 (44%) can be attributed to rather an irreversible structural failure. These showed an inferior mechanical strength owing to the existence of the less structured gel-like system and weakly connected networks.^5,19^ In accordance with these data, the steady and oscillatory rheological tests revealed that a less structured ink showed a weaker elastic property. Therefore, an excellent dynamic network with a desirable reversible structure was fabricated in the Pickering emulsion inks, especially those with a higher ratio of grafted micro-biosurfactant.

### Characterization of 3D-Printed Objects

#### Printing Performance

To perform an effective 3D printing process, a high level of pseudoplasticity and viscoelasticity offers ink high printability.^3,10^ Under this condition, the inks can easily be extruded out during the 3D printing process. Furthermore, a more reversible ink having smaller droplets/particles, which are uniformly dispersed in the emulsion, can offer an effective 3D printing process concerning the shape stability and resolution of the printed structures.^3^ In the present study, the results obtained above already prove that the SGM3 and SGM4 Pickering emulsions can be characterized as forming superior ink in terms of their rheological and structural properties. In these cases, they showed a higher degree of shear-thinning behavior, greater elastic modulus, and a strong thixotropic feature with lower creep compliance. Moreover, as outlined in the Supporting Information, and specifically in Section S.3.1, the SGM3 and SGM4 inks also presented the smallest particle size (also see Figure 3f) with a monomodal particle size distribution (also see Figure 3g) and higher ζ-potential (also see Figure 3h), denoting a more structured Pickering emulsion.

**Figure 4:**
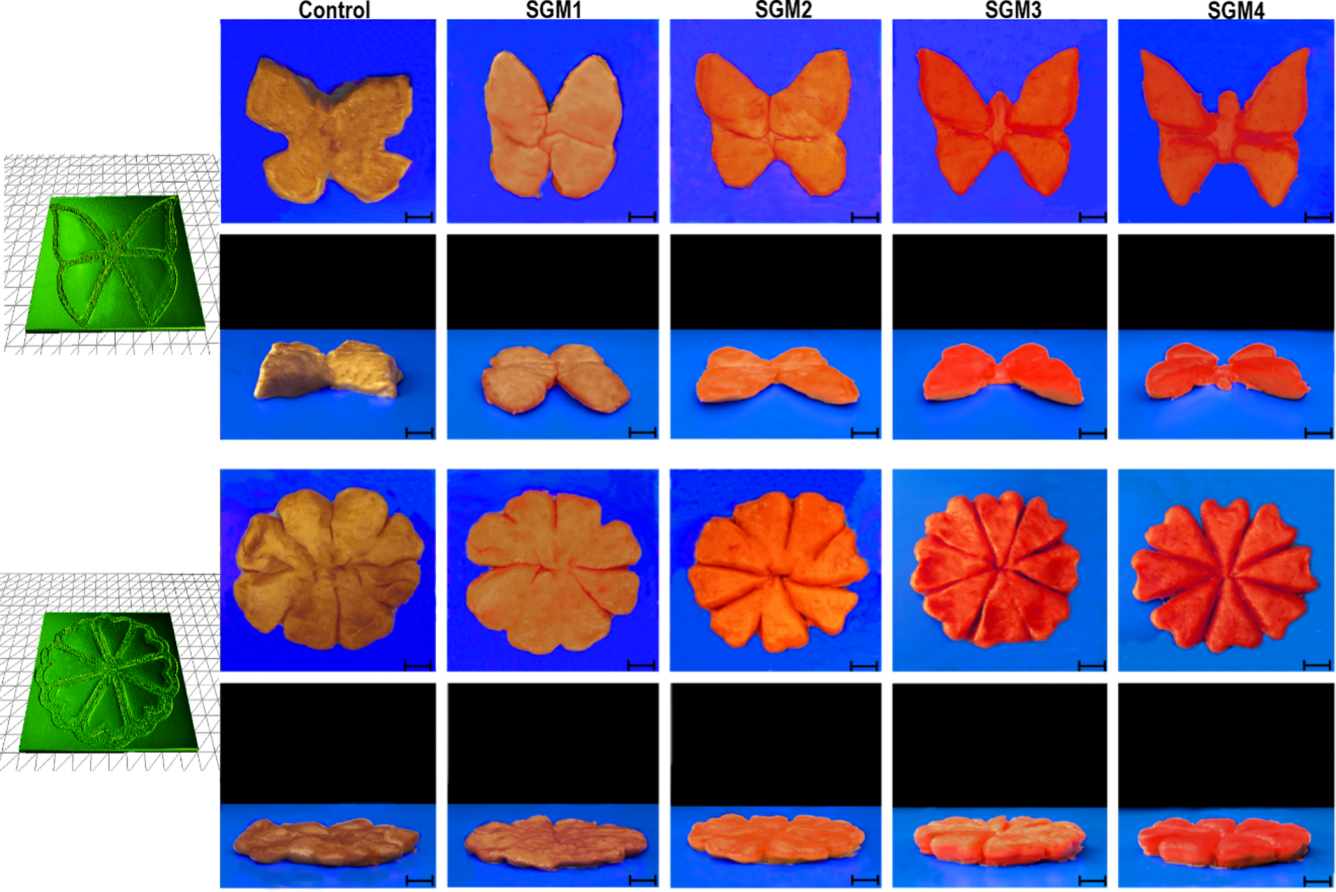
Printing performance photographs of different 3D-printed structures. The scale bar is 5 cm.

Figure 4 illustrates the photographs of 3D-printed soy protein-based architectures containing different ratios of grafted MCC conjugates, which were printed in a layer-by-layer manner. The 3D structures involving SGM4 ink, with 5.6 wt % grafted micro-biosurfactant, preserved their shape during the printing process, while also resulting in a fine resolution with a smooth surface (Figure 4, fifth column). The shape produced with SGM3 ink, having 4.2 wt % grafted micro-biosurfactant, was printed without failure upon the printing process. However, after the printing process, the supporting structure in the underside layer was weakened and somewhat deformed (Figure 4, fourth column). The SGM2 ink, involving 2.8 wt % grafted micro-biosurfactant, was moderately easily extruded out from the nozzle but deformed without keeping the structure at the curved area of the designed shape (Figure 4, third column). Finally, the control (with no grafted micro-biosurfactant) (Figure 4, first column) and SGM1 (with only 1.4 wt % grafted micro-biosurfactant) (Figure 4, second column) were stacked upon printing and then crumbled in the middle of the process. These latter two inks could not provide sufficient support, and the stacking became untenable in the upper portion of the design. As a consequence, it was confirmed that the increasing level of micro-biosurfactant could enhance the layer resolution, leading to a high geometrical accuracy. An increase in the content of grafted MCC/GA/LAE reinforced the mechanical strength of the inks, where the emulsion gels showed a higher structural strength of the internal linkages. This enhances the spatial resolution of the resulting 3D-printed objects.^3,6,10^

To assess the structural strength and printing precision, the layer number (assigned to the structural strength) and line width (related to the printing accuracy) of 3D-printed architectures were evaluated (Supporting Information Table S4). The layer numbers and line widths of the printed control, SGM1, and SGM2 were statistically similar to each other (*P* > 0.05). On the contrary, the printed SGM3 and SGM4 objects offered the 3D structures higher layer numbers and thinner line widths, representing a desired structural strength and well-defined shape. The manufacture of 3D constructs with a precisely controlled structure caused by the inclusion of higher micro-biosurfactant ratios could be elucidated due to the development of a structured matrix with a strong elastic matrix and reinforced connected network. As viscoelastic data show, the SGM3 and SGM4 inks provided a higher value of the elastic property with enhanced structural recovery. This could offer the Pickering emulsion gel resistance against the rapid shear stress and deformation during the 3D printing process.

#### Morphology of 3D-Printed Objects

**Figure 5:**
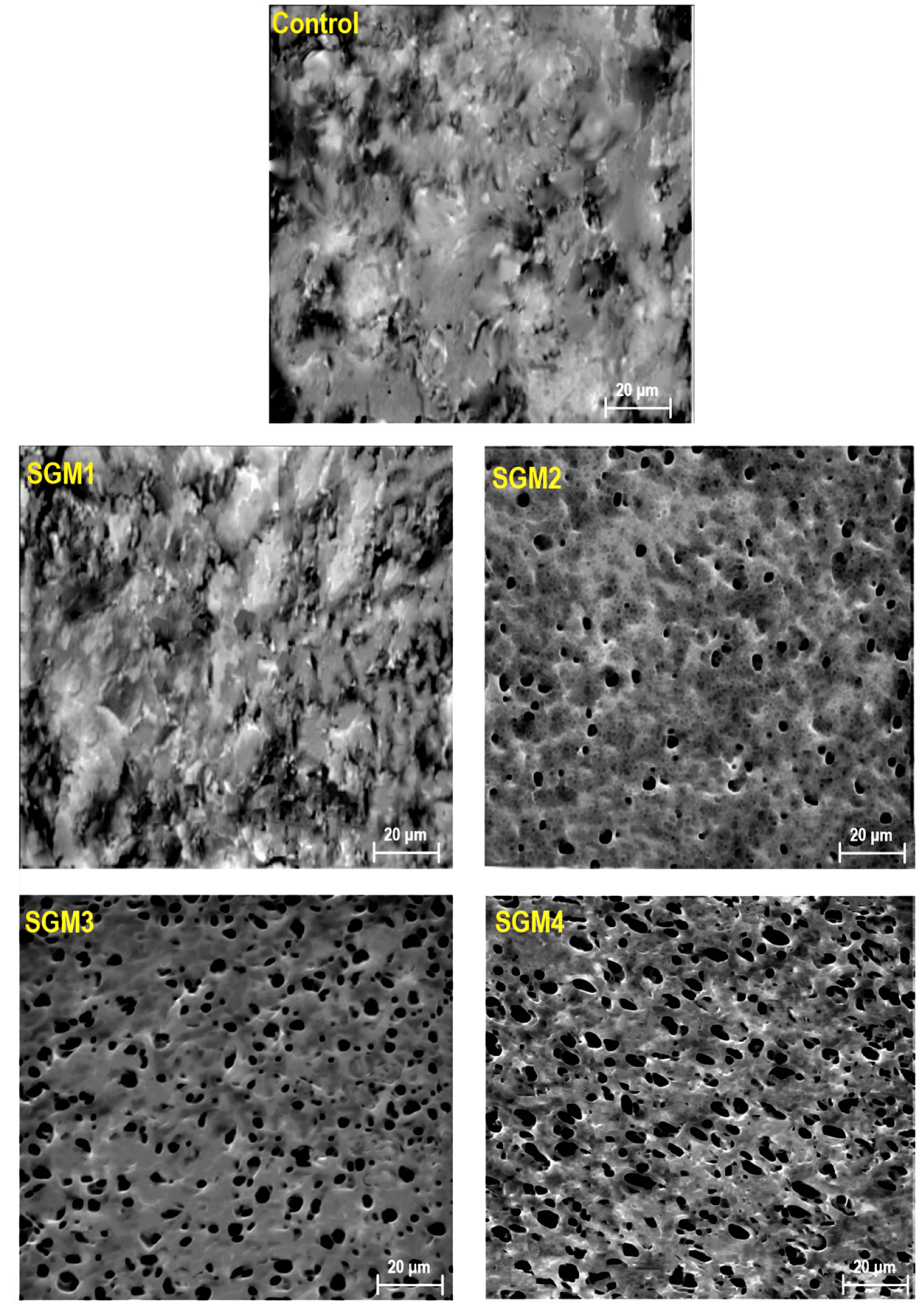
VP-SEM photomicrographs of different meat analogues variants.

Figure 5 shows the VP-SEM microstructure of the printed objects developed with different levels of grafted micro-biosurfactant. The microstructure of printed control seemed to be rugged and irregular with noticeable agglomerated pieces. Besides, there is no obvious pore structure inside its matrix. As Figure 5 depicts, the incorporation of 1.4 wt % grafted micro-biosurfactant could not induce a positive effect on the microstructural properties. This means that the microstructure of printed SGM1 had some level of unevenness with the existence of the aggregated particles on the surface. Moreover, its structure seemed to be denser than that of other reduced-fat printed objects. Regarding printed SGM2, there is the appearance of a limited number of pores with small size and more spherical shape. The VP-SEM micrograph also presented pores in the printed SGM2 randomly dispersed throughout the matrix. In the case of printed SGM3 and SGM4, there was a large number of pores with smaller sizes that were regularly distributed within the matrix. In this context, the highest ratio of grafted MCC conjugate, *i.e*., printed SGM4, could enhance the porosity with an ordered pore, while this was not the case for printed SGM3. Then, the changes in the printing performance and structural stability of 3D structures could be due to differences in their microstructure and therefore the properties.^6^

#### Dynamic Sensory Evolution

**Figure 6:**
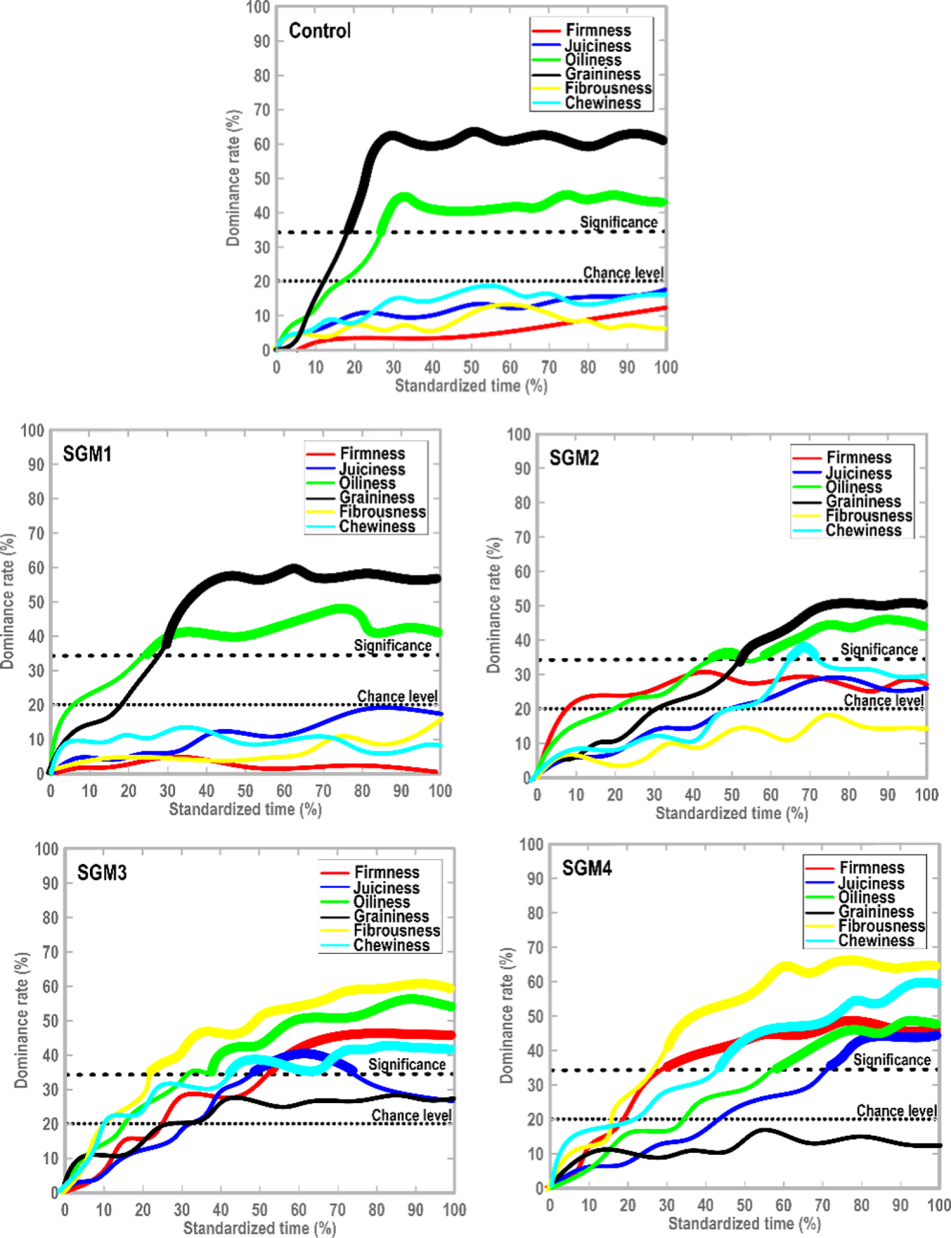
Temporal profile of dominant sensations in standardized time with specific attributes in meat analogue samples.

To accomplish the temporal perceptions of different sensory attributes, the TDS technique is considered an effective tool to compare the sensations perceived simultaneously in a complex food product.^27,28^Figure 6 shows the TDS curves of 3D-printed meat analogue variants, where the significance and chance levels are specified. Based on a binomial distribution and in view of 30 evaluations, the chance (20%) and significance (34%) levels, obtained from six traits, were determined. Figure 6 displays that the graininess trait was a dominant attribute in the 3D-printed control, SGM1, and SGM2 with a maximum dominance rate (max. DR%) of 63.5, 60.1, and 50.9%, respectively. This trait prevailed during the whole period of the sensory evaluation (*P* < 0.05). Based on the VP-SEM experiment, a high level of irregularity with some aggregated micro-sized particles (except SGM2) were detected on the surface of these printed samples. Contrary, the grainy texture in the 3D-printed SGM3 and SGM4 was not perceived as significantly dominant at any time of the sensory assessment (*P* > 0.05). An even and homogeneous structure could be possibly developed in these samples as revealed by microstructure investigations, which showed a smaller pore size distribution with an extremely porous matrix.

In the case of printed SGM3 and SGM4, the chewiness and firmness traits were also dominant at the middle of the consumption time. In the temporal profile of printed SGM2, the chewiness attribute also created a trivial peak, albeit significant, at the middle of TDS evaluation (*P* < 0.05). By referring to the instrumental texture measurement, the printed SGM3 and SGM4 meat analogues proposed a firmer matrix compared to 3D-printed SGM1 and SGM2 (Supporting Information Section S.5.1). These reduced-fat printed samples presented a strong gel-like matrix; thus, they rationally needed a greater force required to chew their matrix. Similarly, the 3D-printed SGM3 and SGM4 meat analogues showed a juicy attribute, especially at the middle of the mastication period, with greater dominance regarding printed SGM4 (max. DR = 45.6%). A promising sensory result was detected for the fibrous attribute, where the printed SGM3 and SGM4 showed the greater dominance of fibrousness with a max. DR of 46.8 and 51.4%, respectively. However, the fibrous sensation in the printed SGM1 and SGM2 was not perceived as significantly dominant throughout the consumption time (*P* > 0.05).

In summary, the newly developed bioactive soy-based Pickering emulsion gel, stabilized by multifunctional microcrystalline cellulose, established feasibility and efficiency for applications in 3D food printing. As gallic acid is well known for its antioxidant ability and lauric arginate shows an interfacial stabilizing effect with excellent antimicrobial activity, we attempted to improve the interfacial activity of microcrystalline cellulose *via* interfering with intra- and intermolecular hydrogen linkages and hydrophobic interaction and also reinforced its antioxidant and antimicrobial activities through grafting of gallic acid and lauric arginate onto the surface of microcrystalline cellulose. After introducing multifunctional microcrystalline cellulose to a soy-based dispersion, a Pickering emulsion gel showing pseudoplastic, viscoelastic, and thixotropic properties was obtained, having a monomodal particle size distribution. The soy protein-based Pickering ink was also effectively processed through an extrusion-type printing system to produce a fibrous meat analogue with a high degree of shape fidelity. The printing performance results, obtained from the inks containing the grafted microcrystalline cellulose, offered the enhancement of layer resolution with a high geometrical precision. A promising result regarding the sensorial properties of the 3D-printed meat analogues was the evidence of a fibrous sensation, which was confirmed by dynamic sensory evaluation. Such introductory results in 3D printing showed how this technique could further generate plant-based meat with the desired texture for enhanced eating experiences. The novel applications in food texture modification to potential fabrication of printable emulsions with a flexible interfacial behavior are promising viewpoints. Considering the obtained results, the food-grade particulate-type inks are not only attractive from an academic look but would have an excessive effect on the construction of suitable emulsion systems in industrial applications.

## Supporting Information Available

^13^C NMR (Section S.1); crystallinity index obtained by ^13^C NMR (Section S.1.1); transmission electron microscopy (TEM) (Section S.2); measurement of droplet size and electrical charges (Section S.3); droplet size and ζ-potential (Section S.3.1); confocal laser scanning microscopy (CLSM) (Section S.4); textural properties (Section S.5); textural evaluation (Section S.5.1); toxicity (Section S.6); printing settings expressed as Slic3r terms (http://slic3r.com) (Table S1); references used to acquaint the panelists with each attribute (Table S2); obtained consistency index, flow behavior index, and yield stress of soy protein-based ink variants (Table S3); and summary of printing performance results and textural properties of 3D-printed meat analogues (Table S4) (PDF)
