# Supplementary material for: A Promising Therapeutic Soy-Based Pickering Emulsion Gel Stabilized by a Multifunctional Microcrystalline Cellulose: Application in 3D Food Printing
Source: J Agric Food Chem. 2022 Feb 10;70(7):2374–88. doi: 10.1021/acs.jafc.1c05644 (PMC8880380; doi:10.1021/acs.jafc.1c05644)
Supplement: Supplementary file 1 — jf1c05644_si_001.pdf [file jf1c05644_si_001.pdf]

Supporting Information for:

A promising therapeutic soy-based Pickering emulsion gel stabilized by a multifunctional microcrystalline cellulose: Application in 3D food printing

Mahdiyar Shahbazi†, Henry Jäger†*, Rammile Ettelaie‡*

†Institute of Food Technology, University of Natural Resources and Life Sciences (BOKU), Muthgasse 18, 1190 Vienna, Austria

‡Food Colloids and Bioprocessing Group, School of Food Science and Nutrition, University of Leeds, Leeds, LS2 9JT, UK

S.1. ^{13}C NMR

The crystallinity index of pristine MCC and grafted MCC conjugate was also taking into account using separating the C4 area of the spectrum into the amorphous and crystalline peaks and determined by dividing the area of the crystalline peak (96 to 102 ppm) by the total area associated with the C4 peak (92 to 102 ppm).

S.1.1. Crystallinity index obtained by ^{13}C NMR

The crystalline and disordered components of unmodified MCC were also calculated in the solid-state ^{13}C NMR spectra as the downfield and upfield lines for the C4 or C6 carbons, respectively. As Figure 2b shown, the unmodified MCC presented a wide non-crystalline resonance cantered at 84 and 62.7 ppm (upfield lines, *i.e*., C4' and C6', respectively). The downfield signals cantered at 92 and 65.3 ppm (C4 and C6, respectively) were assigned to the ordered cellulose structures. The crystallinity index of the unmodified MCC, MCC/GA, and grafted MCC conjugate was then measured using the peak separation method of Newman (2004), by dividing the area of the crystalline peak by the total area assigned to the C4 peaks. The obtained crystallinity index values offered that a little change in the crystallinity index of MCC (~84%) occurred during the grafting treatment (~86%).

S.2. Transmission electron microscopy (TEM)

To evaluate the morphology of the pristine MCC, MCC/GA, and grafted MCC conjugate, the TEM was performed on a Hitachi-7650 TEM (Hitachi Co., Ltd., Japan) at an acceleration voltage of 80 kV. The particle aqueous suspension (0.05 wt%) was placed onto a Formvar-coated 200-mesh TEM grid (Canemco Inc., Canada) and dried at room temperature before observation. The MCC dimensions presented (cross-section and length) were measured from the investigation of a minimum of 50 particles.

S.3. Measurement of droplet size and electrical charges

The inks were diluted to a droplet level of about 0.005 wt.% with a buffer solution at the pH of the inks to avoid multiple scattering impacts. The dispersion was stirred gently at room temperature to ensure the

emulsions were homogenous. The droplet sizes and particle size distribution of the inks were measured with a laser diffraction device (MS2000, Malvern Instruments Ltd., Worcestershire, UK), measuring the size based on the scattering of a monochromatic beam of laser light (k = 632.8 nm). The droplet size was specified as the surface-weighted mean $d_{(3,2)} = (\sum n_i d_i^3 / \sum n i d_i^2)$ and volume means diameters $d_{(4,3)} = (\sum nidi^4 / \sum nidi^3)$, where n is the number of droplets with diameter d_i (Shahbazi et al., 2021). The electrical charges (ζ-potential) of the printable inks were obtained through a Zetasizer Nano-ZS90 (Malvern Instruments, Worcester-shire, UK) at a fixed detector angle of 90°. The Pickering emulsions were diluted to a final concentration of 0.005 wt.% before each measurement to minimize multiple scattering effects. After loading the inks into the chamber of the Zetasizer, they were equilibrated for 5 m before zeta potential data was obtained over 40 continuous readings.

S.3.1. Droplet size and zeta potential

Controlling the particle size is an effective way to meet the requirements of the printable inks for 3D printing purposes. It was stated that a reduction in the particle size of ink dispersions improves the ink functionality in terms of printability and shape-fidelity (Wu et al., 2016; Lee, Won, Kim, & Park, 2019). The droplet sizes of soy-based ink as influenced by different ratios of grafted MCC conjugate were evaluated over time to check its effectiveness on aging (Figure 3f). The results revealed that the $d_{(3,2)}$ and $d_{(4,3)}$ diameters for SGM1 and SGM2 increased after 21 days. In the presence of the low concentration of surface-active MCC, most of the individual droplets, developed through the extreme energy dissipation, were not remained in the final ink, which led to larger droplet sizes (Dickinson, 2003). The phenomenon incidence might be associated with the dissociation of thin-film among the collision droplets (coalescence) or separation of the adsorbed layer between two droplets (flocculation). The flocculation phenomenon is predominant in the concentrated emulsions with the relatively low ratio of emulsifier to oil, and less concentrated systems comprising a mixture of biopolymeric surfactant with different surface activities (Dickinson& Galazka, 1991). Contrarily, SGM3 and SGM4 inks presented smaller droplet sizes compared to SGM1 and SGM2, whose $d_{(3,2)}$ and $d_{(4,3)}$ remained

constant over the 21 days. This showed the coalescence and/or flocculation did not happen in the system. An increase in the viscosity of the continuous phase offered by the higher micro-biosurfactant contents could be justified by the stabilizing effect of grafted MCC conjugate on the soy-based emulsion. Additionally, it was reported the presence of intermolecular interactions between hydrophilic/hydrophobic groups in the system contributes to the biosurfactant effectiveness on aging (Dickinson & Galazka, 1991; Dickinson, 2003).

The particle size distribution (PSD) profiles of soy-based emulsion as affected by the different ratios of micro-biosurfactant are shown in Figure 3g. The PSDs for SGM3 and SGM4 inks were more uniform when compared with SGM1 and SGM2, proposing better emulsion stability. Furthermore, their distribution was monomodal, showing no stable flocs were recognized during the PSD experiment. It could also be revealed the PSD results were in accordance with obtained data for $d_{(3,2)}$ and $d_{(4,3)}$. The improved capacity to reduce the surface/interfacial tension might be due to the presence of more hydrophobic/hydrophilic groups in the system. Accordingly, the grafted LAE on the MCC could mostly interact with oil and more polar cellulose chain ends mainly interacting with the hydrophilic groups. This proposes an important reduction in the interfacial free energy to avoid phase separation and system destabilization. Besides, the reduced droplet size and improved homogeneity with increasing the grafted micro-biosurfactant ratio could be probably owing to an increase in the oil droplets surface coverage, expressing as interfacial biosurfactant concentration. The result of PSD supports the above interpretation that the stabilizing effect of grafted MCC conjugate is either due to an increase in viscosity of the continuous phase and/or the surface activity of the modified MCC.

The zeta (ζ) potential is a physicochemical factor of specific significance in describing the surface electrical properties of charged media, which is a useful parameter for determining electro-kinetic surface features of both intact and modified surfaces. As the electrostatic repulsive force performs as one of the important interactions in hindering the aggregation of biopolymeric emulsion, the ζ-potential of the inks prepared with different ratios of grafted MCC conjugate was obtained (Figure 5h). The ζ-potential of soy-coated droplets regarding control ink was determined to be −14.6 mV (pH 7.0, 0.1 g NaCl), which was expected as the soy

dispersions at pH higher than the isoelectric point (about 4.5) are negatively charged (Lam, Paulsen, & Corredig, 2008). Compared to control, replacement of the lowest level of grafted MCC conjugate (*i.e.*, SGM1) did not change significantly the ζ-potential ($P > 0.05$). However, the ζ-potential of the particles stabilized by 2.8 wt.% grafted MCC conjugate (*i.e.*, SGM2) was increased to −20.4 mV. In this case, the small increase in the ζ-potential (more negative) specified that the steric repulsive forces are comparatively present due to introducing more negatively charged groups into the system. Likewise, the negative ζ-potential more increased when the grafted micro-biosurfactant was incorporated in the ratios of 4.2 (−33.5 mV) and 5.6 wt.% (−44.1 mV). This prevents the development of strong electrostatic repulsive forces regarding SGM3 and SGM4 compared to other Pickering emulsion variants. As the grafted MCC is negatively charged, it is rationally to be expected the negative ζ-potential was more increased as the ratio of grafted MCC was increased. This proposed desirable protection against droplet aggregation owing to a combination of electrostatic and steric stabilization.

S.4. Confocal laser scanning microscopy (CLSM)

The interfacial framework and network structure in the continuous phase of the soy protein-based inks were imaged via an FV-300 confocal laser scanning microscopy system (CLSM, Olympus, Tokyo, Japan) coupled with an Olympus IX71 inverted microscope and an argon-ion laser. The inks (5 mL) were stained with the defined level of Nile Blue A (1.0%, w/v) in deionized water, or the blend of Nile Blue A (1%, w/v) and Nile Red (0.1%, w/v) in 1,2-propanediol (including deionized water, 20 μL g^{-1}) to mark the protein and/or modified MMC and oil droplet, respectively (Shahbazi et al., 2021). The level of both Nile blue A and Nile red solution was 0.01% (w/v). The excitation wavelengths of fluorescent in the system were 488 nm (Nile red) and 633 nm (Nile blue A). The ink microstructures were imaged at ambient temperature directly after staining. All images were obtained at 40× magnification and processed using Olympus Fluoview software (version 2.1, Olympus, Tokyo, Japan).

S.5. Textural properties

The textural parameters of 3D printed meat analogues were determined from a force-deformation plot using a texture analyzer (TA.XT-plus, Stable Micro Systems, Godalming UK). The printed samples were cut into cylindrical shapes that have a dimension of 30 mm in diameter and 10 mm in thickness and then were compressed with a cylindrical probe (75 mm diameter) based on the texture profile analysis (TPA) method (Bourne, 1978). The texture analyzer was adjusted at 5 mm s^{-1} with a compression distance of 8 cm and a peak force of 15 N. The height of the first force peak on the TPA curve is considered as the hydrogel hardness. The cohesiveness was obtained by the ratio of the positive force region after the second compression phase during the first compression phase. The gumminess was determined from the multiplying hardness in the cohesiveness value. The springiness was considered as the height recovered upon the period between the end of the initial compression and the start of the second compression. The chewiness was finally measured as gumminess × springiness. All TPA parameters were measured using the Exponent Lite software (v.6.1.4, TA.XT-plus, Stable Micro Systems, Godalming UK).

S.5.1. Textural evaluation

The texture is a physical feature relating to sensory perception and structural characteristics of materials, which can offer useful information regarding the quality properties and shelf-life of 3D printed products. In this sense, the TPA assay can then support a better understanding of the role of grafted MCC conjugate in the structuring of 3D printed objects. The important textural parameters, including hardness, cohesiveness, gumminess, springiness, and chewiness were determined by the TPA method. The textural experiment showed that hardness (as an indicator of system rigidity) of printed control was slightly increased after the addition of 2.8 wt.% grafted MCC, whose extent from an initial value of 11.2 N significantly increased to 12.4 N ($P < 0.05$) (Table S4). The hardness value was more increased by about 200% regarding printed SGM3 and SGM4. The considerable increase in the hardness parameter of these printed samples might be attributed to the presence of more hydrophobic/hydrophilic groups in the system to initiate the biopolymeric

coils overlap, in which the development of intermolecular junctions could then limit the elongation and arrangement of the chains (McClements, 2015). This could possibly lead to more alignment of the polymeric chains during the formation of 3D structures, producing a more compact and stronger 3D printed network. The TPA test revealed that cohesiveness and gumminess parameters did not affect by the ratios of 1.4 and 2.8 wt.% grafted MCC conjugate ($P > 0.05$), while the grafted micro-biosurfactant at the levels of 4.2 and 5.6 wt.% increased the cohesiveness and gumminess parameters ($P < 0.05$). In the case of springiness (referred to as elasticity), the replacement of grafted MCC conjugate did not significantly change the springiness ($P >$ 0.05), excluding its highest ratio (*i.e.*, SGM4). In this regard, the springiness from an initial value of 4.6 mm increased to 5.1 mm regarding the printed SGM4 object. According to TPA measurement, the chewiness values of printed control, SGM1, and SGM2 were statistically similar to each other ($P > 0.05$). In contrast, the chewiness value meaningfully increased by about 320% regarding printed SGM3. Similarly, a superior increase in the chewiness by about 476% was detected for printed SGM4 (Table S4). The higher values of chewiness concerning 3D printed SGM3 and SGM4 could be ascribed to the strong interaction between hydrophilic groups of modified MCC and soy protein through hydrogen bonding, and the presence of hydrophobic interaction among the LAE portion with oil. This develops the intramolecular linkages between the polymeric chains and consequently strengthening the 3D structure. For the purposes of current work, the 3D printed objects with firmer, more chewable, and strong gel-like structure could be beneficial to consider a desired edible 3D structure, suggesting the advance of instrumental readings for the specific sensory perceptions.

S.6. Toxicity

In the current study, the conformity of the MCC/GA/LAE with the actual food regulations on the food products was verified by cytotoxicity test to study the possibility of utilizing in the food sector. In this regard, the release of cytoplasmic lactate dehydrogenase (LDH) from the incubated cells can be considered as an indicator for

cytotoxicity assay (Shahbazi et al., 2016; Shahbazi et al., 2017). The grafted micro-biosurfactant conjugate did not show any evidence of the cytotoxic effects since they could not increase the cytoplasmic lactate dehydrogenase release from L-929 fibroblast cells in contact with the samples.

The same procedure was performed on reduced fat inks containing MCC/GA/LAE. Similar results were achieved in comparison with the educed fat 3D printed samples containing MCC/GA/LAE. Cytotoxicity result of the reduced fat inks was revealed that the extent of cytotoxicity was less than 5.0% after 3, 18 and 36 h when the ink samples was direct contact with L-929 cells. There was also no significant difference in the release of the LDH compared with the positive control ($P > 0.05$). This indicates that MCC/GA/LAE was non-toxic to the cells.

Apart from these results, numerous studies have been evaluated the antibacterial activity and antifungal properties of MCC containing GA or LAE. The antibacterial activity of MCC containing LAE against E. coli and S. aureus has been reported in the literature. In some studies, the results of the cytotoxicity test also show that the toxic effects of MCC with LAE on cell viability had no significant difference in comparison to P188-SLNs and Tween-SLNs.

References:

Shahbazi, M., Rajabzadeh, G., & Sotoodeh, S. (2017). Functional characteristics, wettability properties and cytotoxic effect of starch film incorporated with multi-walled and hydroxylated multi-walled carbon nanotubes. *International journal of biological macromolecules*, *104*, 597-605.

Shahbazi, M., Ahmadi, S. J., Seif, A., & Rajabzadeh, G. (2016). Carboxymethyl cellulose film modification through surface photo-crosslinking and chemical crosslinking for food packaging applications. *Food Hydrocolloids*, *61*, 378-389.

Table S1. The printing settings are expressed as Slic3r terms (http://slic3r.com).

Printing adjusting	Sign	Value	Units	Definition
**Nozzle diameter**	D	1.0	mm	Nozzle diameter
**Layer height**	Z	1.0	mm	Layer height
**Extrusion flow speed**	Q	0.40	mL min^{-1}	Continuous extrusion flow rate provided by the syringe pump
**Flow rate**	S	90	%	The volume of ink that passes through the extruder
**Infill velocity**	V	15	mm s^{-1}	Spindle speed during extrusion
**Travel velocity**	V_{travel}	180	mm s^{-1}	The spindle speed of a jump between the end of one extrusion and the next
**Perimeter**	P	10	-	Number of outline layers
**Infill density**	ρ_{infill}	90	%	Quantity of material filling the object

Table S2. References used to acquaint the panellists with each attribute.

Attribute group	Attribute	Definition
**Texture and mouthfeel**	Firmness	Effort or force required to bite through the sample
	Juiciness	Describes the amount of liquid released and perceived during mastication
	Oiliness	Describes a feeling of greasy and slippery sensations perceived during mastication
	Graininess	Presence of particles in the mouth, inhomogeneity
	Fibrousness	The extent to which fibres are perceived during chewing
	Chewiness	The total number of chews necessary for food to be ready for swallowing

Table S3. The obtained consistency index, flow behavior index, and yield stress of soy protein-based ink variants.

Samples	Flow behavior index (n)	Consistency index (Pa s^n)	Yield stress (Pa)	R^2
**Control**	0.931±0.010[d]	10.37±0.52[a]	1.96±0.27[a]	0.971
**SGM1**	0.952±0.012[e]	18.31±0.91[a]	2.03±0.55[a]	0.986
**SGM2**	0.824±0.017[c]	29.53±0.54[b]	5.33±0.32[b]	0.985
**SGM3**	0.542±0.015[b]	35.52±1.27[c]	9.44±0.67[c]	0.986
**SGM4**	0.455±0.016[a]	49.75±0.80[d]	11.15±0.66[d]	0.990

[a–e] Means (three replicates) within each column with different letters are significantly different (P < 0.05), Duncan's test.

Table S4. Summary of printing performance results and textural parameters of 3D printed variants.

Sample	Printing performance				TPA parameters				
	Butterfly		Special circle						
	Line width (mm)	Layer number	Line width (mm)	Layer number	Hardness (N)	Cohesiveness	Gumminess (N)	Springiness (mm)	Chewiness (N mm)
**Control**	8.8 ± 0.7[c]	2 ± 1[a]	7.9 ± 0.8[d]	2 ± 1[a]	11.2 ± 0.3[a]	0.38 ± 0.02[a]	4.3 ± 0.4[a]	4.6 ± 0.2[a]	19.6 ± 2.4[a]
**SGM1**	8.6 ± 0.5[c]	2 ± 1[a]	7.8 ± 0.4[d]	2 ± 1[a]	11.1 ± 0.4[a]	0.38 ± 0.02[a]	4.2 ± 0.5[a]	4.4 ± 0.4[a]	18.6 ± 2.4[a]
**SGM2**	8.2 ± 0.6[c]	5 ± 1[c]	6.9 ± 0.5[c]	2 ± 1[a]	12.4 ± 0.2[b]	0.39 ± 0.03[a]	4.8 ± 0.3[a]	4.3 ± 0.6[a]	20.8 ± 2.0[a]
**SGM3**	2.5 ± 0.2[b]	5 ± 1[c]	2.1 ± 0.2[b]	5 ± 1[b]	33.3 ± 0.3[c]	0.42 ± 0.03[a]	14.0 ± 0.5[b]	4.5 ± 0.3[a]	63.0 ± 3.7[b]
**SGM4**	1.6 ± 0.1[a]	8 ± 1[d]	1.3 ± 0.1[a]	8 ± 1[c]	33.9 ± 0.4[c]	0.54 ± 0.03[b]	18.3 ± 0.6[c]	5.1 ± 0.2[b]	93.4 ± 5.8[c]

[a–e] Means inside each column with different letters are significantly different ($P < 0.05$), Duncan's test.

References:

Bourne, M. C., & MC, B. (1978). Texture profile analysis.

Dickinson, E. (2003). Hydrocolloids at interfaces and the influence on the properties of dispersed systems. *Food hydrocolloids*, *17*(1), 25-39.

Galazka, V. B., Dickinson, E., & Ledward, D. A. (1999). Emulsifying behaviour of 11S globulin Vicia faba in mixtures with sulphated polysaccharides: comparison of thermal and high-pressure treatments. *Food Hydrocolloids*, *13*(5), 425-435.

Lam, M., Paulsen, P., & Corredig, M. (2008). Interactions of soy protein fractions with high-methoxyl pectin. *Journal of agricultural and food chemistry*, *56*(12), 4726-4735.

Lee, J. H., Won, D. J., Kim, H. W., & Park, H. J. (2019). Effect of particle size on 3D printing performance of the food-ink system with cellular food materials. *Journal of Food Engineering*, *256*, 1-8.

McClements, D. J. (2015). *Food emulsions: principles, practices, and techniques*. CRC press.

Newman, R. H. (2004). Carbon-13 NMR evidence for cocrystallization of cellulose as a mechanism for hornification of bleached kraft pulp. *Cellulose*, *11*(1), 45-52.

Shahbazi, M., Jäger, H., & Ettelaie, R. (2021). Application of Pickering emulsions in 3D printing of personalized nutrition. Part I: Development of reduced-fat printable casein-based ink. *Colloids and Surfaces A: Physicochemical and Engineering Aspects*, *622*, 126641.

Wu, H., Cheng, Y., Liu, W., He, R., Zhou, M., Wu, S., ... & Chen, Y. (2016). Effect of the particle size and the debinding process on the density of alumina ceramics fabricated by 3D printing based on stereolithography. *Ceramics International*, *42*(15), 17290-17294.
